# Intratumoral Microbiota in Fueling Cancer and Steering Treatment Response

**DOI:** 10.1002/mco2.71052

**Published:** 2026-10-06

**Authors:** Minhui Xu, Jie Yu, Ying Chen, Shengfang Ge, Lin Ye, Yefei Wang, Yixiong Zhou, Renbing Jia

**Affiliations:** ^1^ Department of Ophthalmology Shanghai Key Laboratory of Orbital Diseases and Ocular Oncology Ninth People's Hospital, Shanghai Jiao Tong University School of Medicine Shanghai China; ^2^ Shenzhen Eye Medical Center Shenzhen Eye Hospital, Southern Medical University Shenzhen China

**Keywords:** cancer therapy, carcinogenesis, intratumoral microbiota, microbial targeting, treatment response, tumor microenvironment

## Abstract

Microorganisms within tumors, also known as intratumoral microbiota, actively shape the tumor microenvironment (TME), influencing cancer progression and treatment response. Research has therefore expanded beyond the gut microbiota to intratumoral bacterial and fungal communities. Advances in low‐biomass sequencing, contamination control, and spatial profiling have made it possible to detect these low‐abundance microbes within the TME and map their marked heterogeneity across cancer types and tissue compartments. However, their origins, causal functions, and clinical relevance remain debated. Here, we summarize contamination‐sensitive workflows and high‐resolution techniques required for low‐biomass tissues, and compare representative taxa in major cancer settings. We further explore microbial origins, taxonomic diversity, and localized interactions with malignant, immune, and stromal cells. We synthesize mechanistic evidence linking intratumoral microbes to genomic instability, epigenetic regulation, oncogenic signaling, metabolic reprogramming, and metastasis. Based on these insights, we evaluate their impact on cancer therapies, alongside emerging microbial interventions and barriers to clinical translation. Ultimately, this review provides a framework for assessing intratumoral microbiota as biomarkers, treatment‐response modifiers, and therapeutic targets, while highlighting the key evidence needed to translate these findings into the clinic.

## Graphical abstract

Emerging research underscores the tumor‐specific colonization of microbiota and the role of intratumoral microbiota in cancer development and treatment resistance across multiple malignancies. Here, we provide a systematic review exploring the latest biotechnological advancements for studying low‐abundance intratumoral microbiota, their origins, diversity, and underlying procarcinogenic mechanisms. We also highlight emerging microbe‐associated therapeutic strategies, to bridge the gap between current research findings and clinical applications. Created in BioRender. Xu, M. (2026) https://BioRender.com/qy1gp30.

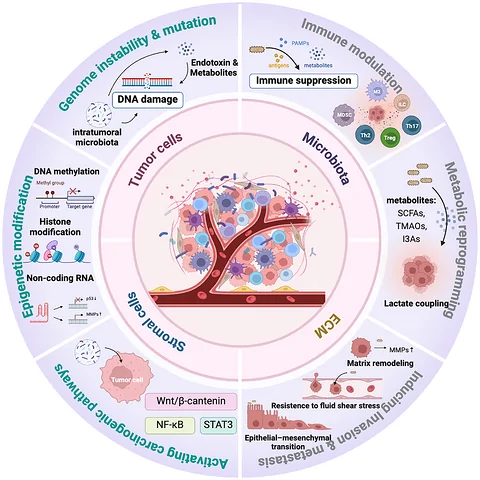

## 1 Introduction

The symbiotic relationship between human microorganisms and their hosts is characterized by mutual dependence and coevolution. Trillions of microorganisms, including bacteria, fungi, and viruses, inhabit various regions of the human body, such as the skin, oral cavity, intestinal tract, respiratory tract, and urogenital system, and play crucial roles in health and disease [1]. Microbiota refers to the consortium of microorganisms that coexist at a specific site and time, whereas dysbiosis describes an imbalance in microbial composition or function that disrupts host homeostasis [1]. Increasing evidence has linked microbiota dysbiosis to a wide range of diseases, including inflammatory bowel disease, multiple sclerosis, Alzheimer's disease, atherosclerosis, diabetes, and cancer [2].

Cancer, a multifactorial disease, arises from a combination of genetic and nongenetic factors, including environmental conditions, diet, lifestyle choices, and infections. Notably, nearly 20% of cancer cases are attributed to infectious agents, which has drawn increasing attention to the relationship between microbiota and carcinogenesis [3]. Early research focused primarily on the gut microbiota and revealed its role in both the risk and treatment of cancer, mainly through its effects on inflammation, systemic immunity, nutrient or drug metabolism, and intestinal mucosal barrier integrity [4, 5, 6]. In recent years, however, advances in next‐generation sequencing (NGS), low‐biomass detection, contamination control, and spatially resolved approaches have enabled the identification of microorganisms in tumors once considered sterile, including those of the breast, pancreas, liver, and prostate [7, 8, 9]. These findings have led to the recognition of intratumoral microbiota as an emerging component of cancer biology.

The tumor microenvironment (TME) is traditionally viewed as a dynamic ecosystem surrounding malignant cells, composed of immune infiltrates, stromal cells, matrix components, and soluble signaling factors that collectively shape tumor behavior and therapeutic response [10]. The intratumoral microbiota comprises bacteria, fungi, and other microorganisms that reside within the TME and is increasingly recognized as an integral part of its ecosystem [7, 8, 9]. Accumulating evidence indicates that the composition, diversity, and function of intratumoral microbiota differ from those of normal adjacent tissues (NATs), suggesting the presence of tumor‐associated microbial dysbiosis [9, 11, 12]. Importantly, while the gut microbiota represents a high‐biomass mucosal ecosystem that influences cancer largely through systemic pathways, the intratumoral microbiota typically has low biomass, may arise through multiple routes such as mucosal barrier breach, hematogenous or lymphatic spread, and adjacent tissue invasion, and appears to exert more localized effects on tumor cells and immune cells within the TME [13, 14]. Despite growing interest, current evidence remains fragmented across tumor types, microbial kingdoms, and methodological platforms, underscoring the need for an integrated review of this rapidly evolving field.

It is also important to distinguish the emerging concept of intratumoral microbiota from the more established paradigm of infection‐driven carcinogenesis. Certain oncogenic viruses, such as Epstein–Barr virus (EBV), human papillomavirus (HPV), and hepatitis B virus (HBV), can induce carcinogenesis through mechanisms including oncogene introduction, inhibition of tumor suppressor genes, DNA integration causing chromosomal instability, and epigenetic regulation [15, 16, 17]. By contrast, with regard to intratumoral bacteria and fungi, despite substantial evidence for their procarcinogenic effects on multiple tumor types, no taxa other than *Helicobacter* (*H*.) *pylori* have been confirmed by the International Agency for Research on Cancer as causes of carcinogenesis [18, 19, 20]. Garrett et al. categorized these procarcinogenic effects into three potential mechanisms: (i) altering the host cell proliferation–death balance; (ii) modulating systemic and/or local immunity; and (iii) influencing the metabolism of host cell products, nutrients, and drugs [21]. These concepts provide an important framework for understanding how intratumoral microbiota may contribute not only to tumor initiation, but also to progression, metastasis, and treatment response.

In this review, we first summarize the current concepts and biotechnologies used to study intratumoral microbiota. We then discuss its origins, diversity, and heterogeneity across cancer types, followed by an overview of its roles in tumor initiation, progression, metastasis, and therapeutic response. Finally, we highlight the emerging diagnostic, prognostic, and therapeutic implications of intratumoral microbiota, with particular emphasis on its potential as a source of biomarkers and as a target or adjunct for microbiota‐modulating strategies. By integrating current evidence across mechanistic, technological, and translational dimensions, this review aims to provide a clearer framework for understanding the complex and context‐dependent roles of intratumoral microbiota in cancer.

## 2 Biotechnologies for Studying Intratumoral Microbiota

The development of analytical technologies has enabled advances in the study of intratumoral microbiota, especially given the extremely low microbial biomass in tumor tissues. Currently, high‐throughput approaches for investigating intratumoral microbiota can be broadly classified into two categories: taxonomic profiling technologies, which are used to define microbial composition, and functional profiling technologies, which aim to reveal microbial activity and host–microbe interactions. Advances in bioinformatics methods also allow microbial signals to be mined from previously generated sequencing datasets of tumor tissues, thereby extending the value of existing cancer genomic resources for intratumoral microbiota research (Table 1).

**TABLE 1:** Overview of major technologies for profiling intratumoral microbiota.

Name	Applications	Strengths	Limitations	References
16S rRNA gene sequencing	Characterization of bacterial communities within TME and comparison across clinical conditions (e.g., tumor progression and metastasis)	Requires minimal DNA input; high accuracy; economically feasible for large cohorts; well‐established analytical workflows	Restricted to bacterial taxa; insufficient resolution at species/strain level; susceptible to amplification bias and background contamination; lacks functional insight	[9]
18S rRNA gene sequencing	Profiling of intratumoral eukaryotic microorganisms (mycobiome)	Enables detection of nonbacterial microbial components	Limited taxonomic resolution; pronounced host interference; incomplete reference annotations	[22]
Internal transcribed spacer (ITS) sequencing		Provides high‐resolution fungal identification (species‐level)	Applicable only to fungi; strong primer‐ and PCR‐related amplification bias; high vulnerability to environmental contamination	[12]
Whole metagenome‐based shotgun sequencing (WMS)	Integrated taxonomic and functional profiling of diverse microbial kingdoms in tumors	Allows species‐ and strain‐level discrimination; acquires genetic information of both microbes and host cells; enables inference of metabolic and virulence pathways	High vulnerability to low microbial biomass; high sequencing depth and cost; complex data processing requirements	[23]
Mass spectrometry‐based proteomics	Functional characterization of host–microbe protein interactions and pathway alterations in tumors	Provides direct protein‐level functional information; captures host response and effector molecules; complements DNA‐ and RNA‐based analyses	Low sensitivity for scarce microbial proteins; strong host protein background; difficult protein‐source attribution; technically demanding sample preparation and analysis	[24]
Metabolomics profiling	Identification of microbiota‐associated metabolites influencing tumor biology and immune responses	Provides functional readouts closest to biological phenotype; reveals host–microbe metabolic interplay	Limited ability to trace metabolites to specific microbes; strong confounding from host metabolism; sensitive to sample handling and batch effects	[25]

*Abbreviations*: 16S rRNA, 16S ribosomal RNA; 18S rRNA, 18S ribosomal RNA; ITS, internal transcribed spacer; WMS, whole metagenome‐based shotgun sequencing.

Amplicon sequencing using NGS platforms, including 16S ribosomal RNA (16S rRNA) sequencing for bacteria and 18S rRNA and internal transcribed spacer (ITS) sequencing for fungi, is widely used to characterize the microbial profiles of mixed samples containing genetic material from both host cells and uncultivable microorganisms [26]. On the basis of the subtle differences in hypervariable regions, microbes can be reliably identified, clustered into different taxonomic groups down to the genus level, and phylogenetically analyzed [27]. However, the low microbial biomass within tumors poses two major challenges: (i) a high ratio of tumor‐to‐microbial DNA results in low PCR amplification efficiency, leading to sampling bias, and (ii) a high risk of contamination from reagents, laboratory environments, and personnel [28, 29]. Recommended practices include aseptic sampling, careful control of workspace, and storage conditions to minimize exogenous DNA introduction [30]. Processing multiple controls alongside study samples, such as sampling blanks, extraction blanks, and library‐preparation blanks, may facilitate the identification of background contaminants [30]. Physical separation of pre‐ and post‐PCR procedures may further reduce cross‐contamination risk [30]. During bioinformatic analysis, examination of sequenced controls and the use of contamination‐aware filtering tools may improve confidence in low‐biomass microbial signals. Depletion of host reads is a crucial approach for overcoming the first challenge and can be performed either before DNA extraction by selectively lysing host cells or after DNA extraction by targeting methylated nucleotides that are more frequent in eukaryotic genomes [31, 32, 33, 34]. SourceTracker and Decontam algorithms have also recently been developed to remove contaminant taxa [35, 36]. Whole metagenome‐based shotgun sequencing (WMS) can distinguish species or strains and provide both taxonomic and functional information by capturing total microbial genomic content [23]. Compared with amplicon sequencing, WMS is more affected by low microbial biomass, limiting its application in tumor research [33]. The PICRUSt2 tool was developed to investigate the functional potential of intratumoral bacteria by mapping 16S sequences to genes and pathways that these species may carry [9].

Research outcomes vary considerably because studies differ in microbial gDNA extraction, hypervariable region selection for amplicon sequencing, sequencing platforms, amplicon NGS analysis workflows (e.g., chimera detection, operational taxonomic unit clustering, taxonomic assignment, reference database selection, and diversity analysis) [28, 37]. The selection of amplicon regions within the 16S rRNA gene is a common source of this heterogeneity because the gene contains nine hypervariable regions (V1–V9) interspersed with 10 conserved regions [38]. Although sequencing the full‐length 16S rRNA gene offers improved taxonomic resolution, short‐read sequencing that encompasses single or multiple hypervariable regions remains a widely adopted method for phylogenetic analysis and taxonomic assignment of human commensal microbiota due to its lower cost [39, 40]. In silico comparisons with full‐length sequencing have generally identified V4 as the preferred single hypervariable region for 16S rRNA sequencing [41, 42]. Primers specific for the V4 or V3–V4 regions are the most commonly used in most cancer types, including CRC, pancreatic cancer, breast cancer, lung cancer, and melanoma, due to their ability to provide relatively high resolution across a broad range of bacterial taxa [8, 43, 44, 45, 46, 47]. Nevertheless, considering that different tumors harbor distinct microbial communities and certain hypervariable regions are more effective at identifying specific taxa, it is crucial to optimize hypervariable region selection for each cancer type [48]. For example, Costantini et al. reported that V3‐region sequencing provides slightly better characterization of the breast microbiota than does V4 sequencing, with greater detection of Actinobacteria and lower detection of Bacteroidetes [38]. V3–V5 region sequencing has previously been reported to perform best for breast tissue [49]. Nejman et al., Narunsky‐Haziza et al., and Dohlman et al. have each conducted comprehensive analyses of bacterial and fungal signatures in multiple cancer types via their respective standardized workflows, thereby uncovering distinct microbial species associated with different malignancies [9, 11, 12]. The amplified regions selected by Nejman et al. comprised five short reads covering the V2, V3, V5, V6, and V8 regions, thereby expanding the taxonomic coverage suitable for human pan‐cancer studies and increasing the resolution to the species level compared with the V4 or V3–V4 regions [9]. Narunsky‐Haziza et al. identified tumor‐specific intratumoral fungal communities and revealed the mycobiome–immune interactions via a combination of ITS2 amplicon, shotgun metagenomic, and metatranscriptomic sequencing [12].

The approaches summarized in Table 1 provide bulk‐level compositional and functional information that, when integrated with single‐cell and spatially resolved technologies, enable a more refined dissection of intratumoral microbiota localization, cellular tropism, and functional niches within the TME. Galeano Niño et al. combined SRT with scRNA‐seq to capture bacterial transcripts with spatial coordinates and host cell barcodes from human CRC and oral squamous cell carcinoma samples [50]. The results revealed that intratumoral bacteria are mostly intracellular and tend to colonize immunosuppressive and poorly vascularized microniches, and that intracellular bacteria can cause chromosomal instability in epithelial cell clusters and affect the expression of host cell genes, such as multiple proinflammatory cytokines, chemokines, and metalloproteinases [50]. Another study based on SRT revealed that bacteria are enriched in tumor cells compared with other immune and stromal cells in early‐stage lung cancer and that the intracellular bacterial load is positively correlated with the expression of oncogenic β‐catenin [51].

Tumor whole‐genome sequencing (WGS) and whole‐exome sequencing (WES) data can also be secondarily leveraged to explore microbial signals by analyzing nonhost reads [52, 53]. Public cancer genomics resources, such as WGS datasets from The Cancer Genome Atlas (TCGA), have accordingly been used to infer the presence of intratumoral microbes [6]. However, because contamination can profoundly distort taxonomic profiles and even generate spurious cancer‐type‐specific signatures, rigorous quality control is essential in such analyses. Recommended precautions include stringent host depletion (particularly high‐copy human sequences, including long interspersed nuclear elements and short interspersed nuclear elements), inclusion of human and vector sequences in reference databases, use of curated contaminant‐screened genomes, and cautious interpretation of low‐biomass signals [6]. Dohlman et al. analyzed the microbial profiles of different cancer types from WGS and WES datasets across 1772 patients and 3698 samples from the TCGA database and developed a statistical method to distinguish tissue‐resident species from contaminants based on their prevalence in tissue and blood [29].

Overall, advances in NGS technology and bioinformatics tools are driving rapid progress in intratumoral microbiota research (Figure 1) [26]. These technical considerations provide an essential foundation for understanding the origins, composition, and biological significance of intratumoral microbiota.

**FIGURE 1:**
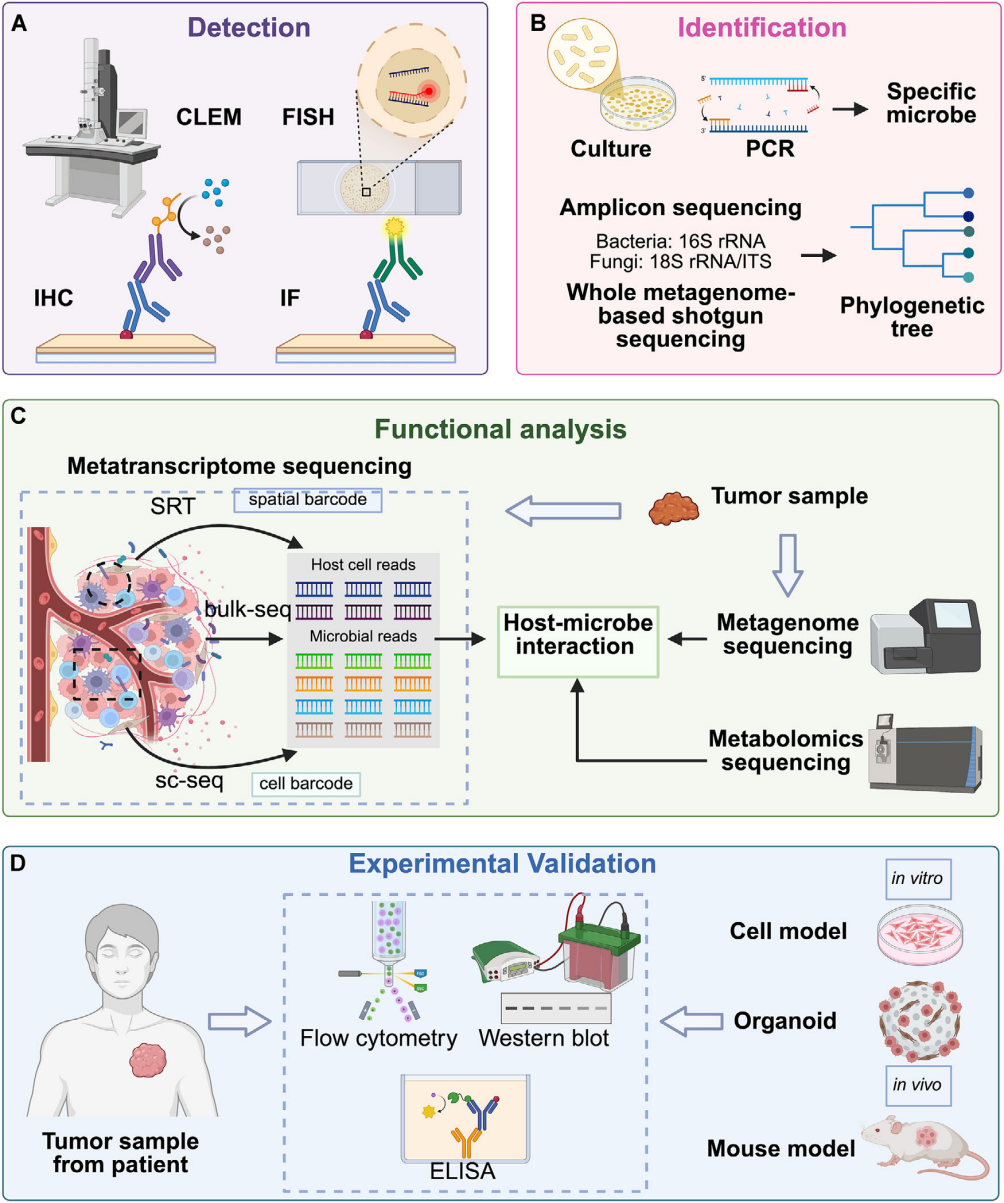
Biotechnologies to study the characteristics and effects of intratumoral microbiota. (A) Detection: correlative light and electron microscopy (CLEM), fluorescence in situ hybridization (FISH), immunohistochemistry (IHC), and immunofluorescence (IF) enable direct visualization of microbes within tumor tissues. (B) Identification: culture and PCR can identify selected known microorganisms, whereas high‐throughput approaches, including amplicon sequencing and whole metagenome‐based shotgun sequencing, profile broader microbial communities. (C) Functional analysis: metagenomic, metatranscriptomic (e.g., bulk sequencing, single‐cell RNA sequencing, and spatially resolved transcriptomics), and metabolomic approaches help characterize host–microbe interactions and associated functional changes. (D) Experimental validation: flow cytometry, western blot (WB), and enzyme‐linked immunosorbent assay (ELISA), together with in vitro and in vivo experiments, are used to test mechanistic relationships between microbes and tumors. Created in BioRender. Xu, M. (2026) https://BioRender.com/u49n328.

## 3 Characteristics of Intratumoral Microbiota

As evidence of intratumoral microbiota has accumulated, its origins have received increasing attention. The possible origins of intratumoral microbiota can be classified into three categories: (i) mucosal barrier invasion, which is common in multiple types of tumors within open cavities and organs, such as pancreatic cancer, colorectal cancer (CRC), lung cancer, and cervical cancer; (ii) hematogenous spread, in which microorganisms from externally exposed cavities and organs are transported through the circulation and colonize the tumor site through disrupted vasculature; and (iii) adjacent tissue invasion, as some studies have shown that the microbiota of tumor tissues is similar to that of normal adjacent tissues (Figure 2) [14]. For example, *Fusobacterium* (*F*.) *nucleatum*, a gram‐negative anaerobic oral commensal bacterium, has been detected in and reported to promote tumor progression in CRC and breast cancer, likely reaching these tumors through hematogenous spread rather than the gastrointestinal route [54, 55]. *F. nucleatum* enters the bloodstream during transient bacteremia, a common occurrence in periodontal disease, and is selectively enriched in CRC and breast cancer tissues through the binding of its surface‐exposed lectin, Fusobacterium autotransporter protein 2 (Fap2), to Gal–GalNAc, a sugar overexpressed on tumor cells [56]. Fardini et al. found that *F. nucleatum* can disrupt cell–cell junctions in vascular endothelial cells by binding its Fusobacterium adhesin A (FadA) to cadherin, thus increasing vascular permeability and facilitating its translocation and colonization at different body sites [57]. Despite differences in the prevalence of specific taxa, the overall microbial profiles of tumors and NATs are relatively similar [9, 11]. Ascomycota and Basidiomycota are the dominant phyla in the intratumoral mycobiome of many cancers [11]. The ratio of Ascomycota to Basidiomycota is relatively high in CRC and low in melanoma, which corresponds to different fungal communities inhabiting the gut and skin, suggesting the potential for adjacent tissue invasion [11]. In addition to the three classic origins described above, a “Trojan horse” route has been proposed in which bacteria can reside inside immune cells and be carried to tumors via lymphatic trafficking through draining lymph nodes [58]. In a preclinical murine melanoma study, gut microbes were observed to translocate from the intestine to the mesenteric lymph nodes, tumor‐draining lymph nodes, and tumors in a dendritic cell (DC)‐dependent manner [58]. Related immune cell‐mediated dissemination mechanisms have been widely studied in nonmalignant infectious disease models but remain to be further validated in the tumor context [59]. Notably, immune checkpoint inhibitor (ICI) therapy may facilitate the translocation of gut microbes to secondary lymphoid organs and thereby enhance antitumor T‐cell immunity [58].

**FIGURE 2:**
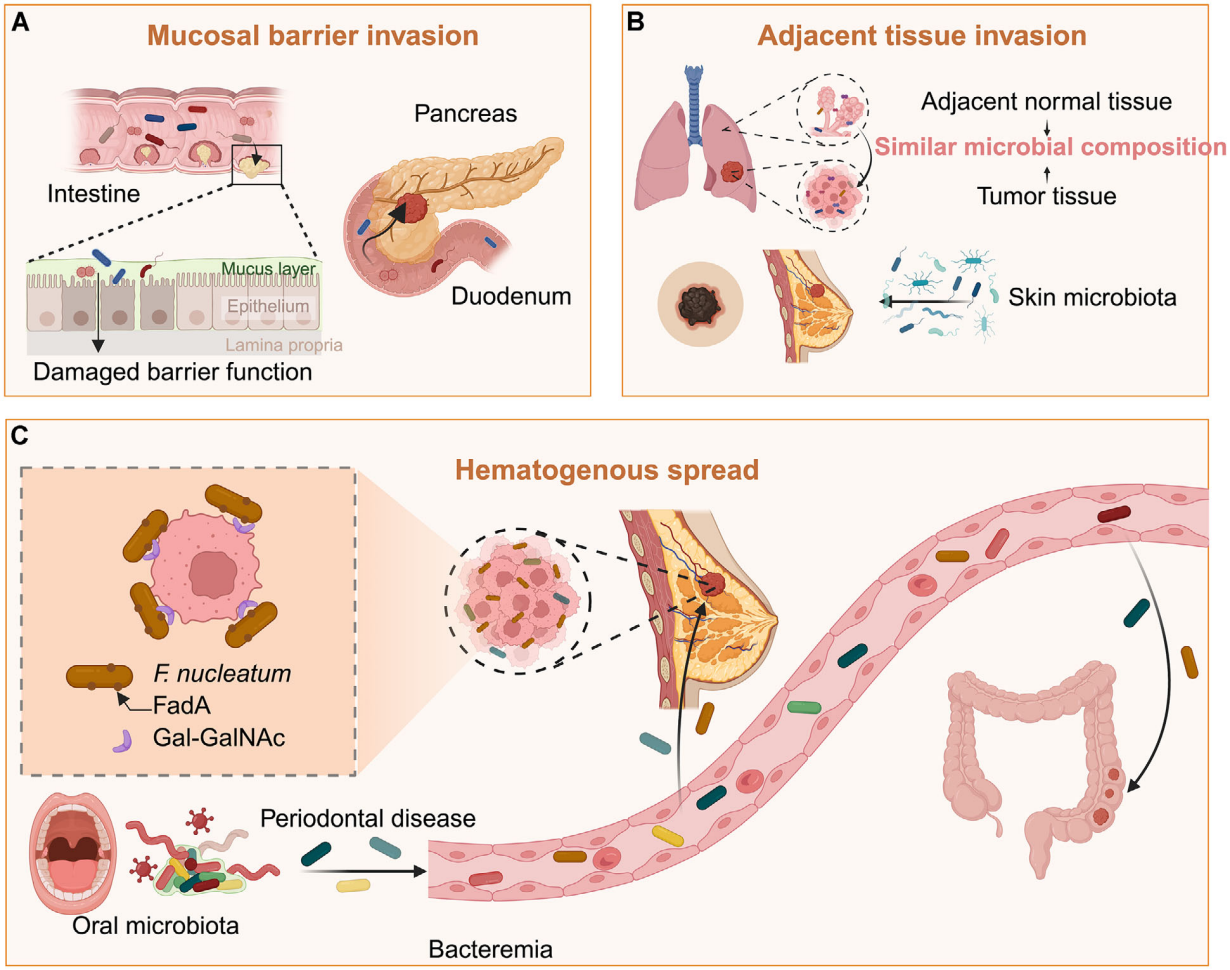
Origins of the intratumoral microbiota. (A) Mucosal barrier invasion: in colorectal cancer (CRC), specific microbial “drivers” can disrupt barrier integrity and facilitate invasion by both driver and passenger microorganisms; in pancreatic cancer, intratumoral microbes may translocate retrogradely from the gut through the pancreatic duct. (B) Adjacent tissue invasion: tumor‐associated microbial communities may resemble those of nearby normal tissues; cutaneous melanoma and breast cancer, for example, show increased representation of skin‐derived microbes. (C) Hematogenous spread: the oral commensal Fusobacterium nucleatum can enter the circulation and selectively enrich in tumors with increased Gal–GalNAc, including CRC and breast cancer, through binding between bacterial surface Fap2 and Gal–GalNAc. Created in BioRender. Xu, M. (2026) https://BioRender.com/e37d827.

Different tumor types harbor diverse microbiological compositions at the phylum, genus, and species levels, which have been uncovered by several comprehensive analyses of a variety of cancer types (Table 2) [9, 11, 12]. Bacteria were confirmed to be more abundant than fungi in the microbial communities of 31 cancer types [12]. Gastrointestinal tumors have a higher abundance of intratumoral microbiota than nongastrointestinal tumors do [11, 29]. Among nongastrointestinal tumors, it has been reported that breast and lung cancers harbor the highest abundance of bacteria and fungi, respectively [11, 29]. Notably, intratumoral bacteria are mainly intracellular, residing within the cytoplasm of tumor cells and various immune cells, especially macrophages [8, 9, 24]. Using correlative light and electron microscopy, Nejman et al. found that intracellular bacteria within breast tumors existed in a wall‐deficient state and close to the nuclear membrane [9]. Similarly, Chai et al. showed lysosome‐encapsulated bacteria in both tumor and peritumoral tissues in intrahepatic cholangiocarcinoma, some of which had been digested [60]. Nevertheless, the proportion of bacteria in different cells remains unclear [8, 9, 24]. Many studies have demonstrated that intracellular bacteria are more enriched in cancer cells than in immune cells, with a cell type‐specific enrichment pattern in which the number of species associated with tumor cells is significantly higher than that in immune cells [8, 51, 60, 61]. In contrast, Li et al. reported that in pancreatic ductal adenocarcinoma (PDAC) and lung squamous cell carcinoma (LUSC), intratumoral bacteria tend to colocalize with immune and stromal cells (approximately 90%) rather than with tumor cells [62]. Bacteria‐derived human leukocyte antigen (HLA) peptides are present in both tumor cells and immune cells in melanoma [24]. These peptides are preferentially presented by certain HLA‐I and HLA‐II molecules over human‐derived HLA peptides and exhibit strong immunogenicity, potentially playing a crucial role in modulating the immune TME [24]. The presence of live bacteria in tumor tissue has been demonstrated by culture [9, 24]. These findings collectively suggest a potential role for intratumoral microbiota and its components and/or metabolites in promoting cancer.

**TABLE 2:** Diverse taxonomic composition of intratumoral microbiota in different tumors.

Cancer type	Microbial composition	Effect	References
Colorectal cancer	Genera *Fusobacterium* and *Bacteroides*	Associated with immunosuppressive and insufficiently vascularized microenvironment	[50]
	*B. fragilis*, *F. nucleatum*, *P. micra*, *Peptostreptococcus stomatis*, and *Gemella morbillorum*	−	[63]
	Enterotoxigenic *Bacteroides fragilis* (ETBF)	The prevalence of *Bacteroides fragilis* toxin (BFT) was higher in late‐stage (III/IV) than early‐stage (I/II) colorectal cancer (CRC) patients.	[64]
	*F. nucleatum*	Related to high microsatellite instability (MSI) and CpG island methylator phenotype (CIMP)	[65, 66]
	*P. micra*	Enriched in tumor tissues; an independent risk factor for shorter survival	[67]
	*Clostridioides difficile*	Derived from patients; promotes carcinogenesis and procarcinogenic mucosal immunity in vivo	[68]
	Phylum Ascomycota; class Saccharomycetes; genus *Candida*; *C. albicans*, *C. tropicalis*	Associated with metastasis, advanced stage, and poor prognosis; promote the expression of proinflammatory cytokines	[29]
Pancreatic cancer	Phyla Proteobacteria, Bacteroidetes, Firmicutes, and Actinobacteria	Induce innate and adaptive immune suppression by activating PRRs	[44]
	Genera *Pseudoxanthomonas*, *Saccharopolyspora*, and *Streptomyces*; *Bacillus clausii*	Associated with high antitumor immune response and long‐term survival	[47]
	Genus *Megasphaera*	Enriched in long‐term survival (LTS) patients; enhance antitumor immune response by producing short‐chain fatty acids (SCFAs)	[69]
	Genera *Finegoldia* and *Nocardioides*	Enriched in LTS patients; trigger lipid peroxidation and ferroptosis by producing butyrate	[70]
	Genera *Acinetobacter, Pseudomonas*, and *Sphingopyxis*	Associated with aggressive “basal‐like” phenotype, shorter survival and higher inflammatory response	[71]
	Genera *Bacteroides*, *Lactobacillus*, and *Peptoniphilus*	Associated with shorter survival and reduced infiltration by CD4^+^ T, CD8*^+^* T, CD45RO^+^ T cells	[72]
	*H. pylori*, *Francisella spp*., and *F. nucleatum*	Colocalized with tumor cells; promote carcinogenesis	[61]
	Class Gammaproteobacteria	Mediate resistance to gemcitabine	[73]
	Genus *Malassezia*	Promote tumor progression by inducing complement cascade	[22]
Breast cancer	Phyla Proteobacteria, Firmicutes, Actinobacteria, and Bacteroidetes; *F. nucleatum*, *L. iners*, *Corynebacterium*, *Streptococcus infantis*, and *Staphylococcus cohnii*	−	[9]
	Phylum Firmicutes; genera *Staphylococcus* and *Lactobacillus*	Promote metastasis by increasing resistance to fluid shear stress	[8]
	Order Clostridiales	Induce pyroptosis of tumor cells by producing TMAO and thereby promoting an antitumor immune response	[25]
	Families Micrococcaceae, Caulobacteraceae, Rhodobacteraceae, Nocardioidaceae, and Methylobacteriaceae; genus *Propionicimonas*	−	[74]
	Genus *Campylobacter*	Associated with increased genomic instability and poor prognosis	[53]
	*F. nucleatum*	Promotes tumor growth and metastasis	[54]
	ETBF	Promotes tumor growth and metastasis	[75]
Lung cancer	Phyla Proteobacteria, Actinobacteria, Firmicutes, and Bacteroidetes; genera *Corynebacterium*, *Sphingomonas yunnanensis*, *Paracoccus marcusii*, *L. iners*, *Roseomonas mucosa*, *Neisseria macacae*, and *Klebsiella pneumonia*	−	[9]
	Genera *Acidovorax*, *Tepidimonas*, and *Brevundimonas*	Enriched in LUSC compared with LUAD	[76]
	Genera *Roseburia*, *Butyricicoccus*, and *Butyricimonas*	Associated with poor recurrence‐free survival (RFS); promote metastasis and immunosuppression via the metabolite butyrate	[77]
	Genus *Escherichia*	Associated with prolonged overall survival (OS) in advanced non‐small cell lung cancer (NSCLC) patients receiving anti‐PD‐1/L1 treatment	[78]
	Genera *Sphingomonas* and *Pseudomonas*	Associated with improved response to chemotherapy combined with immune checkpoint inhibitor (ICI) treatment in NSCLC	[79]
	*L. iners*	Associated with poor recurrence‐free survival (RFS)	[80]
	Genus *Aspergillus*; *A. sydowii*	Associated with poor progression‐free survival (PFS)/overall survival (OS); promotes tumor progression by creating an immunosuppressive TME	[81]
	Order Capnodiales; *Cladosporium sphaerospermum* and *Malassezia globosa*	−	[12]
Cutaneous melanoma	Phyla Firmicutes, Bacteroidetes, Proteobacteria, and Fusobacteria; *Paracoccus marcusii*, and *S. aureus*	−	[9]
	Genus *Lachnoclostridium*	Promotes antitumor immune responses	[82]
	*Malassezia globosa*	−	[12]
Uveal melanoma	Phylum Proteobacteria; genera *Stenotrophomonas*, *Acinetobacter*, and *Pseudomonas*	−	[83]
Liver cancer	Genera *Akkermansia* and *Methylobacterium*; *Akkermansia muciniphila*	Associated with prolonged OS and RFS after surgery	[84]
	Genera *Pseudomonas*, *Thermomonas*, and *Paraprevotellaceae*	Associated with long‐term survival	[85]
	Phyla Proteobacteria and Actinobacteria	−	[84, 85]
	*Paraburkholderia fungorum*	Enriched in NATs; inhibits tumor progression by regulating metabolism	[60]
Esophageal cancer	Genera *Streptococcus*, *Lactobacillus*, *Prevotella*, and *Fusobacterium*	−	[86]
	Genus *Streptococcus*	Enriched in patients responsive to neoadjuvant chemoimmunotherapy; enhances antitumor immune responses	[87]
	*F. nucleatum*	Associated with poor overall survival (OS); contributes to an aggressive phenotype through upregulation of chemokines (e.g., CCL20)	[88]
	*F. nucleatum*	Associated with poor recurrence‐free survival (RFS) and poor response to neoadjuvant chemotherapy	[89]
	*Porphyromonas gingivalis*	Associated with low survival rates	[90]
	*Porphyromonas gingivalis*	A risk factor for local recurrence after endoscopic submucosal dissection (ESD) treatment	[91]
Ovarian cancer	*Gemmata obscuriglobus*, *Halobacteroides halobios*, and *Methyloprofundus sedimenti*	−	[92]
	Phyla Proteobacteria and Firmicutes	Modulate immune TME	[93, 94]
	Genus *Cladosporium*	−	[93]
Cervical cancer	*L. iners*	Confer resistance to chemoradiation through lactate‐induced metabolic coupling	[80]
Bladder cancer	Genus *Oscillatoria*; *E. coli*	Associated with epithelial–mesenchymal transition (EMT)	[95]
Thyroid cancer	Genera *Sphingomonas* and *Aeromonas*	Increased in tumor tissue; discriminate tumor from peritumor tissue	[96]
	Genera *Pseudomonas*, *Rhodococcus*, *Ralstonia*, *Acinetobacter*, and *Sphingomonas*	Associated with increased invasion	[97]
Head and neck squamous cell carcinoma	Genera *Capnocytophaga*, *Fusobacterium*, and *Treponema*	Enriched in tumor tissues and associated with stage	[43]
	Genus *Leptotrichia*	Associated with increased overall survival rates	[98]
	Genus *Fusobacterium*	Associated with increased inflammatory response and poor prognosis	[99]
	*F. nucleatum*	Associated with reduced recurrence and metastatic relapse rates, and prolonged survival	[100]
Glioblastoma/glioma	Phyla Proteobacteria and Firmicutes; families Enterobacteriaceae, Bacillaceae, and Mycobacteriaceae	Recognized by tumor‐infiltrating lymphocytes; suggest a potential role for microbial antigens in antitumor immune activation through cross‐reactivity	[101]
	Phyla Firmicutes and Fusobacteria	Associated with synaptic activity	[102]
	*F. nucleatum*	Promotes glioma growth by modulating immune TME	[102]
	*Staphylococcus warneri*, *Microbacterium esteraromaticum*, *Enterobacter cloacae*, and *Acinetobacter baumannii*	−	[103]
Brain metastases	Genus *Paracoccus*	Associated with poorer overall survival	[103]
	Orders Lactobacillales and Burkholderiales; genus *Rubrivivax*; *Carnobacterium viridans*	Enriched in bacterial metabolic pathways associated with tumor spread and metastasis (e.g., polyamine biosynthesis pathways and the mevalonate pathway)	[103]

*Abbreviations*: BFT, *Bacteroides fragilis* toxin; CCL20, C–C motif chemokine ligand 20; CIMP, CpG island methylator phenotype; EMT, epithelial–mesenchymal transition; ESD, endoscopic submucosal dissection; ETBF, enterotoxigenic Bacteroides fragilis; LTS, long‐term survival; LUAD, lung adenocarcinoma; MSI, microsatellite instability; NSCLC, non‐small cell lung cancer; PD‐1, programmed cell death protein 1; PFS, progression‐free survival; PRRs, pattern‐recognition receptors; RFS, recurrence‐free survival; SCFAs, short‐chain fatty acids; TMAO, trimethylamine N‐oxide.

The crosstalk between tumor cells, immune cells, and intratumoral microbiota is increasingly understood. During microbial colonization and coevolution within tumors, the microbial genome may undergo adaptive changes. For example, exosome‐like nanoparticles derived from lung adenocarcinoma (LUAD) could be internalized by *Staphylococcus* (*S*.) *aureus*, which is enriched in tumors compared with NAT tissues, leading to morphological changes and reduced adhesive capacity and virulence of *S. aureus* [104]. In cervical cancer, *L. iners* acquired an additional *lacG* gene before or during carcinogenesis, whose encoded protein facilitated the conversion of lactose to glucose and galactose‐6‐P, thus increasing lactate utilization [80]. In human PDAC and LUSC, bacterial accumulation was found to be directed by CD8+ T cells, likely mediated by changes in nutrient and metabolite delivery due to vascular alterations [62]. Furthermore, the extracellular matrix (ECM) within the TME affects the intratumoral microbiota. In PDAC, the extensive accumulation of abnormal Type I collagen (Col1) homotrimers (α1/α1/α1) produced by tumor cells due to epigenetically mediated suppression of *Col1a2* gene expression not only induced proliferative signaling through integrins but also altered the microbial composition within tumors, characterized by increased *Bacteroidales* and decreased *Campylobacterales*; this alteration is associated with a suppressed immune response and accelerated tumor progression [105].

Together, these observations suggest that intratumoral microbiota is not a static bystander, but a dynamic and context‐dependent component of the tumor ecosystem. Such complexity provides the basis for examining how intratumoral microbiota may influence tumor initiation, progression, metastasis, and treatment response.

## 4 Effects of Intratumoral Microbiota on Cancers

The initiation and progression of cancer are rarely attributed to a single pathological microorganism, but more generally to global perturbations of the microbial community. Their effects on the TME are context specific: some microorganisms promote tumor progression, whereas others inhibit it [106]. *H. pylori* is the first and only bacterium found to have a causal link with human gastric carcinogenesis even in the absence of previously mutated genes [20]. Nevertheless, other microbes have been implicated in the occurrence and development of gastric cancer. Yue et al. found that in addition to *H. pylori*, *Kytococcus sedentarius*, and *Actinomyces oris* also strongly interact with the host cell genome and induce aberrant DNA methylation, particularly in genes associated with immune or carcinogenic pathways, in gastric adenocarcinoma [107]. Moreover, a clinical study involving 276 gastric cancer patients not receiving chemotherapy showed that the abundance of *H. pylori* was significantly lower in tumor tissues than in normal tissues, whereas the abundances of *Prevotella melaninogenica*, *Streptococcus anginosus*, and *Propionibacterium acnes* were elevated [108]. In summary, the procarcinogenic effects of intratumoral microbes can be categorized into several mechanisms: the induction of genome instability, DNA mutation, and epigenetic modification, and the activation of carcinogenic pathways in tumor cells; and the modulation of the inflammation, immunity, and metabolic reprogramming within the TME (Figure 3) [21]. Due to the limited research available thus far, which has primarily focused on a few cancer types—lung cancer, breast cancer, CRC, and PDAC—the following sections will provide a detailed discussion of the role of intratumoral microbiota in these four cancer types, as well as in metastatic cancer.

**FIGURE 3:**
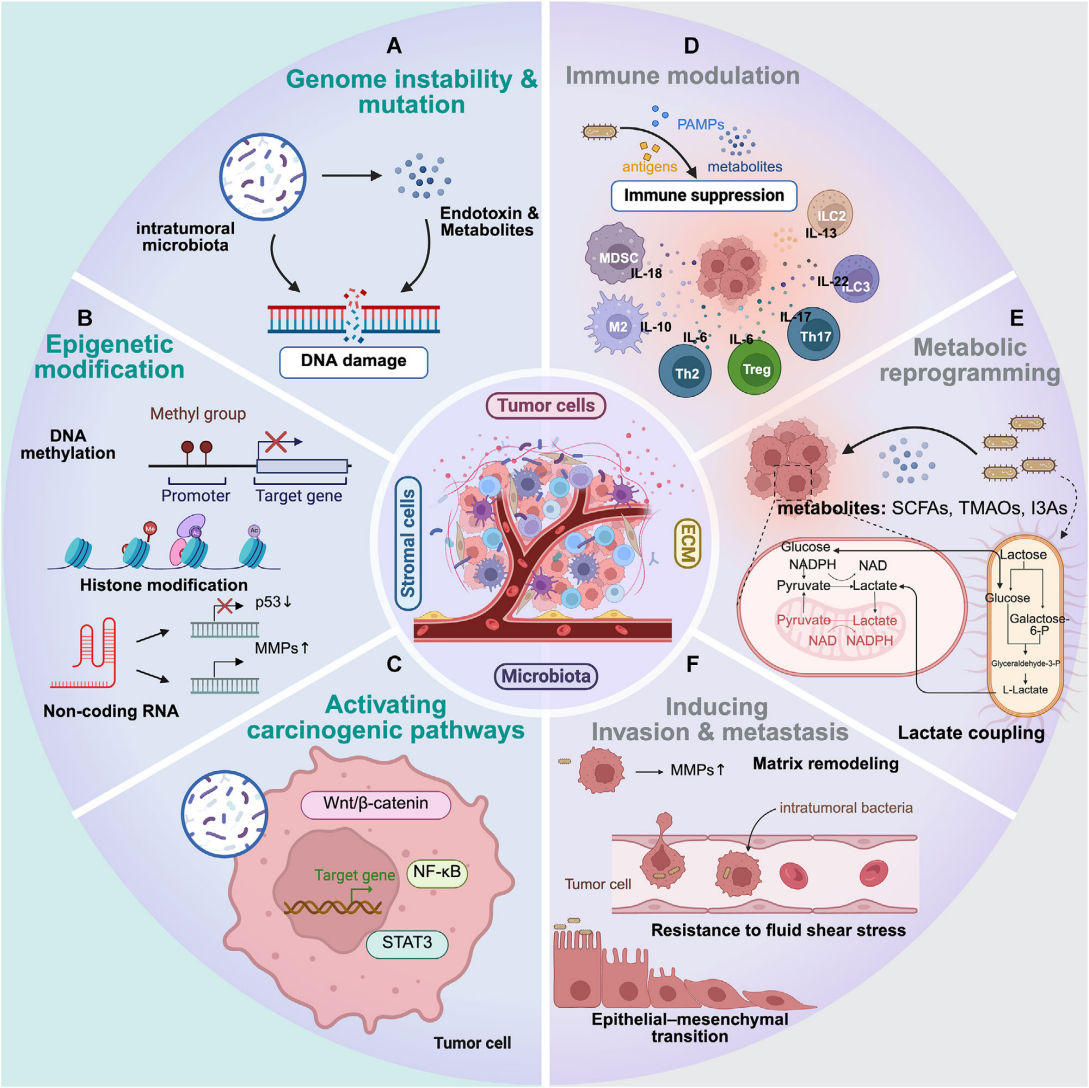
Mechanisms by which intratumoral microbiota promote carcinogenesis and development. Intratumoral microbiota may contribute to cancer development through multiple, interconnected mechanisms. These include: (A) genome instability and mutation, through microbial toxins, endotoxins, and metabolites that induce DNA damage; (B) epigenetic modification, including alterations in DNA methylation, histone modification, and noncoding RNA regulation; (C) activation of carcinogenic signaling pathways, such as Wnt/β‐catenin, NF‐κB, and STAT3; (D) immune modulation, by reshaping the tumor immune microenvironment toward immune suppression; (E) metabolic reprogramming, through microbiota‐associated metabolites and lactate‐related metabolic coupling; and (F) induction of invasion and metastasis, including extracellular matrix remodeling, epithelial–mesenchymal transition, and enhanced resistance to mechanical stress. Together, these processes promote tumor growth, progression, and therapeutic resistance. Created in BioRender. Xu, M. (2026) https://BioRender.com/j82w188.

### 4.1 Lung Cancer

Several small, cross‐sectional cohort studies have reported inconsistent lung cancer dysbiosis signatures across saliva, sputum, airway brushing, bronchoalveolar lavage fluid, and tumor samples from patients and healthy individuals [76, 109, 110]. This diversity makes the microbiota a potential screening biomarker to distinguish lung cancer patients from healthy individuals and predict prognosis, such as metastasis, recurrence, and survival [109, 111]. Nevertheless, the airway microbiota does not precisely reflect the lung microbiome because of potential contamination by oral and upper respiratory tract microbiota [112].

In patients with lung cancer, the microbial loads are higher in tumors and airways than in NATs, indicating that airways may be a potential source of intratumoral microbiota in addition to blood [9, 51]. Proteobacteria, Actinobacteria, Firmicutes, and Bacteroidetes are the most prevalent and abundant phyla in both tumors and paired normal tissues from patients with lung cancer, whereas tumors harbor a significantly reduced microbiome diversity compared with remote normal tissues [9, 76, 112]. At the family level or lower, reported lung cancer‐associated microbial dysbiosis patterns vary widely, which may be attributed to high histological heterogeneity and exposure to various environmental factors (e.g., smoking, air particulates, population density, and antibiotics) [112, 113]. A recent meta‐analysis attempted to generalize the findings and demonstrated a significant reduction in the relative abundance of *Corynebacteriaceae* and *Halomonadaceae* families, and *Corynebacterium*, *Lachnoanaerobaculum*, and *Halomonas* genera in lung tumors compared with NATs [114]. LUSC and LUAD are the most common pathological subtypes of lung cancer with different taxonomic patterns of intratumoral microbiota [76, 113]. Although the 10 most abundant genera are similar in LUSC and LUAD, the microbiome diversity is significantly higher in LUSC than in LUAD [115]. Greathouse et al. showed that *Acidovorax, Tepidimonas*, and *Brevundimonas* were more enriched in LUSC than in LUAD, and within the LUSC subtype, *Acidovorax* was more abundant in *Tp53*‐mutant tumors and samples from smokers [76]. Tobacco smoking, a prominent risk factor for lung cancer, can damage the epithelial barrier and create an environment more susceptible to various pathogens [116]. Metabolic pathways associated with the degradation of tobacco chemicals, such as nicotine, toluene, anthranilate, phenol, and phenolic compounds, are significantly enriched in the intratumoral bacteria of current smoking patients with non‐small cell lung cancers (NSCLCs) compared with never‐smoking patients [9]. Additionally, some biosynthesis pathways of plant metabolites are more enriched in smokers, indicating that some tobacco‐resident microbes might colonize the lung [9]. Microorganisms with either or both of these two functional activities are found mainly in the phyla Proteobacteria, Actinobacteria, and Cyanobacteria, but rarely in Firmicutes and Bacteroidetes [9].

The microbiota and its components or metabolites are associated with lung cancer prognosis. Peter et al. found that both recurrence‐free survival (RFS) and disease‐free survival are positively correlated with overall microbial diversity in remote normal lung tissues of patients, whereas neither outcome was correlated with diversity in tumor samples [117]. Ma et al. showed that the intratumoral microbiome in lung cancer patients with early recurrence (RFS < 3 years) has significantly lower diversity than that in patients with late recurrence (RFS > 3 years), with significantly enriched butyrate‐producing bacteria (e.g., *Roseburia*, *Butyricicoccus*, and *Butyricimonas*) [77]. Moreover, using a combined model of differentially expressed genes and intratumoral microbiota, the accuracy of predicting metastasis, recurrence, and survival in patients with lung cancer could be improved [115, 118]. Interestingly, since lung specimens are not readily available, circulating microbial DNA signatures may provide a promising noninvasive approach for predicting lung cancer recurrence and survival, showing great prognostic value [77, 119, 120].

The procarcinogenic effects of the lung microbiota are mediated mainly by the induction of local chronic inflammation and the modulation of the immunosuppressive TME. Lung‐resident γδ T cells play a key role in coordinating innate immune responses against pathogenic microorganisms via pattern recognition receptors (PRRs), constituting a second line of defense and maintaining lung homeostasis [121]. In a spontaneous LUAD mouse model, pulmonary microbiota dysbiosis was found to trigger interleukin (IL)‐1β and IL‐23 production by myeloid‐lineage cells, inducing local proliferation and maturation of lung‐resident IL‐17‐producing γδ T (γδ T17) cells; increased levels of IL‐17A contributed to increased neutrophil infiltration, causing local inflammation and promoting tumor growth [122]. Intriguingly, IL‐17 and IL‐17‐producing T cells can play distinct roles in different tumor types, mediating both antitumor and protumor functions [123, 124]. In terms of lung cancer, the frequencies of IL‐17‐producing CD4+ T (Th17) cells, γδ T17 cells, and IL‐17 in the peripheral blood were increased in patients with LUAD compared with healthy controls and were correlated with tumor invasion and metastasis [125]. Also, high expression of IL‐17 in NSCLC tissues was reported to be correlated with TNM stage, lymph‐angiogenesis, and poor survival [126]. These results collectively suggest that local inflammation mediated by IL‐17A may promote the initiation and progression of lung cancer. Notably, since γδT17 cells were the major source of IL‐17A and the abundance and maturation of Th17 cells in tumor or tumor‐bearing lung tissues did not differ from those in normal controls in an LUAD mouse model, γδ T17 cells may play a more important role in microbiota‐induced IL‐17 mediated tumor progression than Th17 cells do [81, 121]. Bacterial metabolites may facilitate the immunosuppressive TME; for instance, butyrate at a low concentration was reported to promote the M2‐polarization of macrophages, which secrete IL‐10 and IL‐13, thus promoting tumor growth and metastasis [77]. Intratumoral mycobiome enrichment and dysbiosis have also been observed in lung cancer in humans [81]. In particular, the viable oral taxon *Aspergillus* (*A*.) *sydowii* had a significantly higher abundance in tumors than in nontumor tissues, and increased intratumoral A. sydowii loads were associated with immunosuppression and poor prognosis, characterized by increased myeloid‐derived suppressor cells (MDSCs), regulatory T cells (Tregs), and programmed cell death protein 1 (PD‐1)^+^CD8^+^ T cells within tumors, as well as advanced stage, lymph node invasion, overall survival (OS) and progression‐free survival, respectively [81]. In vivo mechanistic studies using spontaneous LUAD KP mice showed that *A. sydowii* alone could modulate the immunosuppressive TME by promoting the differentiation of myeloid cells toward neutrophilic MDSCs and subsequent IL‐1β secretion via the activation of the β‐glucan‐induced Dectin‐1/CARD9 pathway [81, 127].

In addition to modulating the TME, there is evidence for a direct effect of the lung microbiota on tumor cells. Using spatial meta‐transcriptomic methods, it was found that the majority of intratumoral bacteria are intracellular, with tumor cells harboring the most abundant bacterial burden in lung cancer [9, 51]. The bacterial burden in tumor cells is positively associated with oncogenic signaling pathways, such as Wnt/β‐catenin and hypoxia‐inducible factor‐1α pathways, and negatively modulates molecules affecting the cell cycle, such as TP73 [51]. Microbiota‐induced γδ T17 cells upregulate the expression of IL‐22 and amphiregulin in the lung, both of which can activate the epithelial growth factor receptor and induce tumor cell proliferation [122]. Butyrate, a bacterial metabolite enriched in lung tumor tissues in patients with short RFS, could inhibit p53 signaling and promote the expression of various matrix metalloproteinases (MMPs), thereby promoting tumor progression and metastasis in lung cancer [77, 128]. Notably, butyrate classically exerts anticancer effects as a histone deacetylase (HDAC) inhibitor by inducing apoptosis (e.g., upregulating Bax and downregulating Bcl‐2). In lung cancer, it paradoxically reverses this program via the H19–miR‐675‐5p axis, where p53 suppression leads to Bax inhibition and Bcl‐2 upregulation; mechanistically, this context‐specific effect at relatively low concentrations is attributed to selective inhibition of HDAC2 rather than pan‐HDAC activity, preferentially activating protumorigenic epigenetic programs such as H19 promoter acetylation instead of broad anticancer pathways [77, 128].

### 4.2 Breast Cancer

The microbiota within breast tumors is highly abundant and diverse, potentially originating from the skin, gut, and oral cavity [8, 9, 74, 129]. A comprehensive study of seven cancer types showed that the bacteria in breast tumors were the most enriched and diverse, with an average of 16.4 species per sample, compared with less than 9 in other types [9]. Proteobacteria, Firmicutes, Actinobacteria, and Bacteroidetes are the four predominant phyla in human breast tumors; at the species level, *F. nucleatum*, *Lactobacillus* (*L*.) *iners*, *Corynebacterium US_1715*, *Streptococcus infantis*, and *Staphylococcus cohnii* constitute the major components [9, 130]. Fungi were also detected in human breast tumors, and Ascomycota was the most abundant [131]. Urbaniak et al., Costantini et al., and Nejman et al. all showed that the bacterial profiles of normal NATs and tumors were similar [38, 132]. This similarity suggests that intratumoral microorganisms may migrate to peritumoral tissue, or that dysbiosis in the breast may predate carcinogenesis and modulate the microenvironment prone to tumorigenesis. Nevertheless, the level of microbial abundance in tumors and NATs remains a matter of debate. Nejman et al. showed that both the species richness and bacterial abundance are highest in breast tumors, followed by NATs and normal breast tissue [9]. In contrast, Xuan et al. found reduced bacterial abundance in breast tumors compared with matched NATs, which can be explained by the role of microbiota in maintaining the host immune response in normal breast tissue [133].

Given that breast cancer is highly heterogeneous with four subtypes according to the expression status of the estrogen receptor (ER), progesterone receptor (PR), and human epidermal growth factor receptor (EGFR) 2, the unique signatures of intratumoral microbiota in different subtypes have attracted attention. Remarkable differences in the prevalence of bacterial, fungal, viral, and parasitic taxa have been shown between subtypes, and some specific microorganisms, whose presence or absence can distinguish the subtypes, may serve as biomarkers for diagnosis and prognosis [9, 131]. Metabolic activities may play a role in the selective enrichment of microorganisms [9]. Mycothiol biosynthesis is a significantly enriched bacterial metabolic pathway in ER+ breast tumors [9]. As previous studies have confirmed the higher level of oxidative stress in ER+ tumors compared with ER− tumors and that mycothiol could serve as a detoxifier of reactive oxygen species in bacteria, the upregulation of this metabolic pathway suggests an altered intratumoral microbiota with a greater capacity to adapt to the ER+ TME [134, 135]. Arsenic is a common Class I carcinogen, and exposure to arsenic could promote the transition of well‐differentiated breast cancer cells (ER+/PR+) to invasive basal‐like cells [9, 136]. The enrichment of arsenate detoxification in the microbiota of ER+ subtypes can be explained by the ability to reduce arsenic levels and maintain the differentiated phenotype of breast cancer cells [9].

The role of intratumoral microbiota in promoting breast tumor progression has been widely confirmed by reductionist approaches. *F. nucleatum* could promote both tumor growth and metastasis in an orthotopic mammary tumor mouse model, mainly by suppressing the antitumor immune response and increasing the expression of MMP9, which is associated with invasion, metastasis and angiogenesis in tumors [54, 137]. Consistent with studies of *F. nucleatum* in CRC, it could migrate hematogenously from the oral microbiota and become enriched in breast tumors through the lectin Fap2, whose ligand Gal–GalNAc is overexpressed in breast tumors compared with NATs in humans and mice [54, 137, 138]. Parida et al. found that intratumoral enterotoxigenic *Bacteroides* (*B*.) *fragilis* (ETBF) might migrate from the gut microbiota owing to its high invasiveness, and ETBF colonizing both the mammary gland and gut could induce tumor growth, as well as local and distant metastasis (e.g., lung and liver) via the activation of E‐cadherin/β‐catenin and Notch signaling pathway [75]. *B*. *fragilis* toxin (BFT) played a key role in carcinogenesis, as breast cancer cells displayed a phenotype of increased stemness and invasiveness following BFT exposure in both in vitro and in vivo xenograft models [75]. The *pks+ Escherichia* (*E*.) *coli* strains were able to damage DNA, as evidenced by increased DNA double‐stranded breaks in HeLa cells after coculturing *pks+ E. coli*, but in vivo evidence is lacking [132]. Fu et al. further revealed the mechanism of bacteria‐mediated breast tumor metastasis by establishing a Matrigel organoid model in vitro [8]. Some unique taxa of the intracellular microbiota, such as *Staphylococcus* and *Lactobacillus*, could facilitate stress fiber reorganization and relieve contractile forces via inhibition of RhoA and ROCK, thereby increasing the mechanical resistance of tumor cells to fluid shear stress [8]. These microbes were enriched in the circulating tumor clusters, upregulated the fluid shear stress pathway of tumor cells, and increased the number of metastatic foci in the lungs in vivo [8]. Interestingly, this study demonstrated that the ablation of intratumoral microbiota with retained gut microbiota influenced only the metastatic process, and no significant difference was found in tumor growth, whereas ablation of intratumoral and gut microbiota could suppress both tumor growth and metastasis, indicating the different roles of intratumoral and gut microbiota [8].

In addition, microbial metabolites have been shown to enhance the antitumor immune response in breast cancer [25]. For instance, in triple‐negative breast cancer (TNBC), Clostridiales and their metabolite trimethylamine N‐oxide (TMAO) were found to be enriched in the tumor tissues of an immunomodulatory subtype to a greater extent than other subtypes [25, 139]. In an orthotopic TNBC mouse model, TMAO could induce Caspase 3 and gasdermin E (GSDME)‐mediated pyroptosis of tumor cells by initiating endoplasmic reticulum stress, thereby enhancing the antitumor immune response and the efficacy of anti‐PD‐1 immunotherapy [25].

### 4.3 Colorectal Cancer

Extensive research over the past decades has established a strong association between gut microbiota dysbiosis and the initiation and progression of CRC and has led to the development of several noninvasive diagnostic models using bacterial, fungal, archaeal, and/or viral taxa as biomarkers [5, 140]. Even in precancerous‐stage adenomas, gut dysbiosis can be observed, for example, the enrichment of *Lachnoclostridium* spp., *F. nucleatum*, and *Clostridium hathewayi*, suggesting that microbial dysbiosis may be an early event in CRC development [141]. Regarding the intratumoral microbiota, *Fusobacterium* (∼50%) and *Bacteroides* are the dominant genera in CRC [50]. Interestingly, there may be an anticorrelated coabundance relationship between the “Fusobacterium cluster” and “Bacteroides cluster,” and a shift toward the former is significantly related to colorectal neoplasms [29]. Likewise, Okuda et al. showed that in a total of 29 patients with CRC, the genus *Fusobacterium* was enriched in tumor tissues, whereas the genus *Bacteroides* was enriched in NATs [142]. A meta‐analysis revealed that at the species level, *B. fragilis*, *F. nucleatum*, *Parvimonas* (*P*.) *micra*, *Peptostreptococcus stomatis*, and *Gemella morbillorum* were substantially enriched in CRC compared with normal colorectal tissue [63]. The phylum Ascomycota constitutes ∼80% of the fungal communities, with the class Saccharomycetes accounting for ∼60% of the total fungi [29]. The genus *Candida* and the species *Candida* (*C*.) *albicans* are the most abundant and prevalent at different taxonomic levels, with the proportion of *Candida* positively correlated with the expression of proinflammatory‐associated genes, making the *Candida*‐to‐*Saccharomyces* ratio a valuable prognostic indicator [11]. Additionally, *C. albicans* and *C. tropicalis* are significantly increased in metastatic Stage IV colon cancer, which may be attributed to their association with the downregulation of cellular adhesion pathways and impaired epithelial integrity [11].

There is also heterogeneity in the microbial community within tumors, with the abundance of some known CRC‐associated taxa such as *Fusobacterium*, *Bacteroides*, and *Parvimonas* highly variable between biopsies from the same tumor [143]. Advances in spatial transcriptomics have helped to further reveal the heterogeneous distribution of microbes and to characterize microbe–host cell interactions [50]. Bacteria tend to colonize highly immunosuppressive and insufficiently vascularized microniches in CRC tumors, characterized by myeloid cell enrichment, upregulation of the immune checkpoint protein cytotoxic T‐lymphocyte‐associated protein 4 (CTLA‐4), and downregulation of smooth muscle actin [50]. Moreover, a significant reduction in the expression of the tumor suppressor p53 was detected in such bacteria‐negative regions, suggesting that intratumoral bacteria may play a role in cancer cell transformation [50].

In addition to cross‐sectional studies, mechanistic studies using reductionist approaches support the procarcinogenic role of *B. fragilis* and *F. nucleatum* in CRC pathogenesis (Figure 4) [144]. *B. fragilis* is a commensal microorganism of the human colon, while a subset of strains, termed ETBF, acquire a pathogenicity island encoding BFT, a zinc‐dependent metalloprotease toxin [64, 145, 146]. BFT exposure levels were significantly higher in CRC patients than in healthy controls and were higher in late‐stage (III/IV) than in early‐stage (I/II) disease [64]. BFT binding to colonic epithelial cells (CECs) induces cleavage of the zonula adherens protein E‐cadherin, triggers the Wnt‐β‐catenin signaling pathway, and increases the expression of the proto‐oncogene protein C‐MYC, consequently promoting the proliferation of CECs [64, 147, 148]. In cancer stem cells, ETBF could upregulate the expression of Nanog homeobox (NANOG) and sex determining region Y‐box 2 (SOX2), both of which are crucial for maintaining stemness [149]. Increased expression of NANOG resulted from the demethylation of histone H3 lysine 9 trimethylation (H3K9me3), driven by ETBF‐induced upregulation of histone demethylase 2B (JMJD2B) via Toll‐like receptor 4 (TLR4)–nuclear factor of activated T cells 5 (NFAT5) signaling [149]. The carcinogenicity of BFT was confirmed using antigen‐presenting cell (APC)‐mutant mice, as hyperproliferation of CECs and the IL‐17‐dependent inflammatory cascade of both CECs and immune cells were observed in mice with ETBF colonization [150, 151, 152]. IL‐17, mainly produced by Th17 cells, γδ T17 cells, and innate lymphoid cells (ILC) 3, could facilitate microbe‐induced activation of the NF‐κB pathway in tumor cells, thus contributing to the recruitment of MDSCs and the development of an immunosuppressive TME [150].The oral commensal *F. nucleatum* is rarely seen in the healthy colon; in CRC patients, an increased abundance of *F. nucleatum* was detected in tumor tissue compared with matched NATs, and was rarely detected in fecal samples, which can be explained by its adherent property [153]. Several studies have demonstrated that *F. nucleatum* levels are positively correlated with microsatellite instability (MSI) and CpG island methylator phenotype (CIMP) in CRC patients [65, 66]. *F. nucleatum* could enhance spontaneous tumorigenesis, from aberrant crypt foci and adenoma to adenocarcinoma in APC^Min/+^ mice and tumor progression in xenograft mice [55, 138]. Unlike ETBF, *F. nucleatum* has no known toxins but expresses two outer membrane proteins, FadA and Fap2 [138, 154]. FadA could promote proliferation of tumor cells by activating the E‐cadherin/β‐catenin pathway [138]. The NF‐κB signaling pathway, induced by *F. nucleatum* through activation of TLR4/MyD88 during carcinogenesis, could promote proliferation and inhibit apoptosis of tumor cells [155, 156, 157]. Recently, an ADP‐heptose‐like metabolite of *F. nucleatum* was found to activate NF‐κB signaling through activation of alpha‐kinase 1 (ALPK1)/TIFA signaling pathway, and subsequently upregulate the expression of two antiapoptotic genes, *BIRC3* and *TNFAIP3* [158]. In addition, *F. nucleatum* is involved in establishing an immunosuppressive TME. The infiltration and expansion of MDSCs and M2‐like tumor‐associated macrophages (TAMs), as well as reduced cytotoxic capacity of lymphocytes and natural killer (NK) cells were observed after *F. nucleatum* treatment [55, 159]. Activated NF‐κB signaling could induce the secretion of IL‐8 and chemokines (e.g., C–X–C motif chemokine ligand [CXCL] 1) in tumor cells, contributing to the establishment of a proinflammatory microenvironment [160]. *F. nucleatum* could enhance the Th17 response through its immunoregulatory metabolites, short‐chain fatty acids (SCFAs) [161]. Furthermore, coculture of CRC epithelial spheroids with *F. nucleatum* in vitro changed cell migration from collective to individual, with alterations in the signaling pathways related to remodeling the ECM, cell adhesion, and migration, which is in line with the previous finding that *F. nucleatum* can be detected in distant‐site metastases [50, 162]. Increased secretion of IL‐8 and CXCL1 could induce migration of CRC cells in vitro [160]. Other bacteria, including *P. micra* and *Clostridioides difficile*, were also found to promote carcinogenesis through the induction of tumor cell proliferation and IL‐17 production, with underlying mechanisms similar to that of ETBF and *F. nucleatum* [67, 68].

**FIGURE 4:**
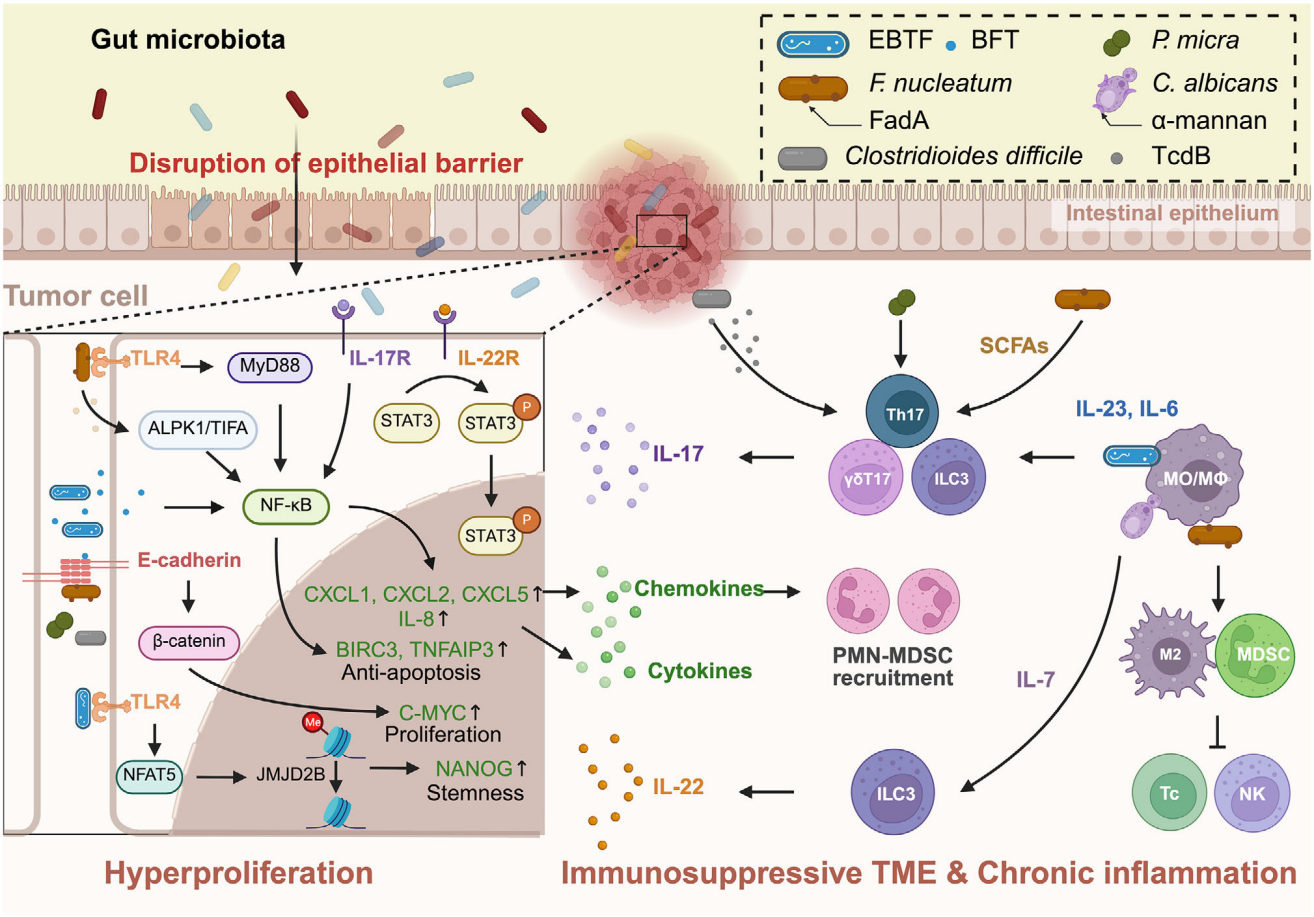
The intratumoral microbiota promotes tumorigenesis in CRC. The main mechanisms include tumor‐cell hyperproliferation, an immunosuppressive TME, and chronic inflammation. FadA expressed on the surface of *F. nucleatum* and BFT secreted by ETBF can induce hyperproliferation of tumor cells by activating E‐cadherin/β‐catenin signaling. In cancer stem cells, ETBF can upregulate the expression of NANOG and SOX2, crucial transcription factors for stemness. The increased expression of NANOG resulted from the demethylation of H3K9me3, driven by ETBF‐induced upregulation of JMJD2B via TLR4–NFAT5 signaling. *F. nucleatum* can activate the NF‐κB signaling pathway by binding to TLR4 or through its metabolite, an ADP‐heptose‐like molecule, thus promoting proliferation, inhibiting apoptosis of tumor cells, and inducing the release of chemokines and cytokines. *Abbreviations*: ETBF, F. nucleatum, *P*. *micra*, and *Clostridioides difficile* can induce an IL‐17‐dependent inflammatory cascade, with *F. nucleatum acting through its SCFA metabolites* and *Clostridioides difficile* by its toxin TcdB. IL‐17, mainly secreted by Th17, γδ T17, and ILC3 cells, can facilitate microbe‐induced activation of the NF‐κB signaling pathway. Activated NF‐κB signaling increases the expression of cytokines (e.g., IL‐8) and chemokines, thus promoting inflammation and recruitment of immunosuppressive MDSCs, respectively. *F. nucleatum* can suppress antitumor immune responses, characterized by M2‐like TAMs, MDSCs, and reduced cytotoxic capacity of lymphocytes and NK cells. *C. albicans* can trigger IL‐22 expression by ILC3, and IL‐22 subsequently induces the phosphorylation of STAT3 and proliferation of tumor cells. Although illustrated using CRC as a representative context, the depicted pathways (e.g., TLR–NF‐κB axes) may operate across tumor types with context‐ and tissue‐specific variations. Created in BioRender. Xu, M. (2026) https://BioRender.com/g95r261.

Fungi have also been implicated in CRC progression. C‐type lectin receptor (CLR) Dectin‐3 plays a key role in recognizing C. albicans through its surface‐exposed α‐mannans and in inducing innate and adaptive immune responses [163]. In CRC patients with high fungal loads, Dectin‐3 and IL‐22 expression is reduced, and Dectin‐3 levels are inversely correlated with the cancer stage, which may explain why *C. albicans* is most abundant in Stage IV CRC patients [164]. An increased abundance of *C. albicans* was observed in a Dectin‐3−/− mouse model, and *C. albicans* can reprogram macrophage metabolism toward glycolysis and subsequently induce the release of IL‐7, triggering the expression of IL‐22 in ILC3s and thus promoting CRC development [164]. Notably, Li et al. provided evidence that cross‐kingdom fungal–bacterial cooperation may contribute to the development and progression of CRC. Specifically, *C. albicans* hyphae were found to facilitate the penetration of *F. nucleatu*m across the intestinal mucosal barrier through a Flo9–RadD interaction, thereby increasing intratumoral colonization of *F*. nucleatum and amplifying its tumor‐promoting effects as described above. Clinical analyses showed that patients with higher fecal levels of *C. albicans* exhibited significantly greater abundance of *F. nucleatum* in tumor tissues than patients with low or undetectable fecal *C. albicans* [165]. These findings highlight that fungal–bacterial interactions, rather than single microbial taxa alone, may be important determinants of intratumoral microbial ecology and oncogenic activity in CRC (Figure 4).

Nevertheless, it is still difficult to study the influence of the intratumoral microbiota in isolation without interference from the gut microbiota because of their dynamic interplay: the gut microbiota may seed intratumoral communities, and both compartments can modulate the immune microenvironment.

### 4.4 Pancreatic Cancer

Several clinical and preclinical studies have demonstrated the substantial abundance of intratumoral microbiota in PDAC, with increases of ∼1000‐fold for bacteria and ∼3000‐fold for fungi compared with those in normal pancreatic tissue [44, 73]. The bacterial and fungal compositions are also distinct from those of normal pancreatic tissue, exhibiting decreased microbiome diversity; 13 phyla of bacteria were detected in the tumors of PDAC patients, with Proteobacteria (∼50%), Bacteroidetes, and Firmicutes being the most dominant; the fungal community of PDAC tumors was significantly enriched for genus *Malassezia* [22, 44]. A single‐cell analysis to investigate the host–microbiota interactions in human PDAC found that host cell‐associated bacteria were present in only 44% of PDAC samples, despite bacteria being detected in 87% of samples [61]. The most common bacteria were *Campylobacter* spp., followed by *F. nucleatum*, *Leptotrichia* spp., and *Clostridioides difficile*, all of which are associated with inflammatory or malignant diseases, while neither fungi nor viruses were detected [61]. Moreover, an SRT study of human PDAC revealed that the majority (∼90%) of bacteria were present in the peritumor cell stroma and colocalized with CD8^+^ T cells [62]. Due to the focal and heterogeneous distribution of CD8^+^ T cells, the tumors can be divided into CD8^+^ T cell‐enriched or deficient areas, referred to as hot or cold nests, respectively [62]. Compared with those within cold nests, the CD8^+^ T cells within hot nests orchestrate the host response to bacteria, resulting in bacterial enrichment with higher abundance and diversity [62]. Notably, PDAC usually progresses rapidly, with a 5‐year OS rate of only 9% [166]. The patient outcome may be associated with the intratumoral microbiota, as the intratumoral microbiome of PDAC patients with long‐term survival (LTS) showed marked differences from that of patients with short‐term survival (STS), whereas no significant host genomic difference was observed [47]. Moreover, patients with LTS had more neoantigens and tumor‐infiltrating cytolytic CD8^+^ T cells, and some of these neoantigens are homologous to noncancer microbial antigens [167]. The prevalence of *Fusobacterium* spp. was associated with a higher rate of cancer‐specific mortality [168]. In addition, Guo et al. found a distinct microbial composition in the invasive basal‐like subtype of PDAC compared with other subtypes, characterized by an increased abundance of *Acinetobacter*, *Pseudomonas* and *Sphingopyxis*, and the abundance of these three taxa was negatively correlated with survival in all PDAC patients [71]. Based on the anatomical position of the tumor, PDAC can be classified into pancreatic head cancer and pancreatic body/tail cancer (PBTC), with PBTC having a poorer prognosis; their differences in microbial composition and associations with clinical manifestations have attracted increased attention (NCT06147154).

Migration from the gut to the pancreas is one of the origins of intratumoral microbiota, as orally gavaged, fluorescently labeled microorganisms were observed to appear in pancreatic tumor tissue in mice [44, 47, 169]. This gut‐to‐pancreatic tumor migration may be taxon‐specific, for example, Proteobacteria, the most abundant intratumoral bacteria, make up only 8% of the gut microbiota; at the genus level, *Pseudomonas* and *Elizabethkingia* were the most abundant within tumors, whereas *Prevotella* and *Bacteroides* were highest in the gut [44]. Considering the anatomical relationship between the pancreas and gut, especially the duodenum, intratumoral microbiota may translocate retrogradely through the pancreatic duct [73]. In humans, bacterial counts were increased in patients with PDAC who received pancreatic duct instrumentation compared with those who did not [73]. Other proposed routes include mesenteric venous drainage and/or mesenteric lymph nodes; these routes have yet to be fully investigated in pancreatic cancer models [170]. Moreover, the intratumoral microbiome clustered separately from that of not only normal pancreatic tissue but also matched gut, and the taxonomic composition of gut microbiota represented only about 25% of the matched intratumoral microbiota in humans, suggesting other potential origins besides gut [22, 47].

In addition to epidemiological associations, intratumoral microbiota have also been confirmed to promote pancreatic oncogenesis in multiple mouse models, such as slowly progressive *p48^Cre^;LSLKras^G12D^* (KC) mice and aggressive orthotopic models with tumor cells derived from *Pdx1^Cre^;Kras^G12D^;Tp53^R172H^* (KPC) mice; and ablation of either bacteria or fungi could protect against oncogenic progression [44, 72, 73]. Modulation of the immune TME is the focus of related mechanistic studies [22, 44, 47, 73]. Greater infiltration of CD3+ and CD8+ T cells and granzyme B+ (GzmB) cells was found in tumors of patients with LTS than in those with STS, and the extent of immune infiltration and activation exhibited a positive correlation with the α‐diversity of intratumoral microbiota [47, 72]. Pushalkar et al. showed that intratumoral bacteria induced both innate and adaptive immune suppression by activating select PRRs, such as TLR2, TLR4, and TLR5 in PDAC mice [44]. In the orthotopic KPC model, microbial ablation led to a significant reduction in MDSCs and an increase in M1‐like TAMs, which mediated enhanced Th1‐polarization of CD4^+^ T cells, as well as activation of cytotoxic CD8^+^ T (Tc) cells in the TME [44]. The activation of TLR4 in immune cells induced T‐cell exhaustion along with increased T cell infiltration and activation in the TME [171]. It was found that lipopolysaccharide (LPS) accumulation within PDAC could activate the TLR4/MyD88/AKT/NF‐κB cascade in tumor cells, promoting immune tolerance and increased expression of programmed death‐ligand 1 (PD‐L1) [171]. In addition, Das et al. showed that intratumoral microbiota‐mediated activation of TLR4 on tumor cells and subsequent involvement of NLRP3 inflammasome could induce the secretion of tumor cell‐derived IL‐1β, which is necessary for the transformation of pancreatic stellate cells to secretory‐phenotype cancer‐associated fibroblasts (CAFs), resulting not only in stromal fibrosis but also in the establishment of an immunosuppressive TME, characterized by M2‐TAMs, MDSCs, and Th17 cells [172]. Fungi regulate the immune TME and promote PDAC primarily by altering innate immunity [22, 173]. Alam et al. found that intratumoral fungi binding to a PRR, Dectin‐1, facilitated the secretion of IL‐33 from pancreatic cancer cells, which enhances the recruitment and activation of Type 2 immunocytes, such as innate ILC2s and Th2 cells, and promotes tumor progression [173]. Aykut et al. reported the procarcinogenic role of fungi by initiating the lectin pathway of complement cascade via mannose‐binding lectin (MBL) pattern‐recognizing carbohydrate ligands on the surface of fungal pathogens [22]. Previous studies have suggested a potential procarcinogenic mechanism in which C3 complement cascade products, C5a and C3a, are released into the TME and recruit MDSCs, inhibit the function of CD8^+^ tumor‐infiltrating lymphocytes, and promote tumor growth and metastasis by initiating an autocrine loop [174]. Insight into the role of specific individual microbes in carcinogenesis is still limited. Udayasuryan et al. showed that *F*. *nucleatum* can bind to and invade PDAC tumor cells in a Fap2‐dependent manner and induce the secretion of granulocyte–macrophage colony‐stimulating factor (GM‐CSF), IL‐8, CXCL1, and macrophage inflammatory protein (MIP)‐3α, which stimulate the proliferation and migration of tumor cells through autocrine and paracrine mechanisms in vitro [175].

Some metabolic products of microorganisms influence carcinogenesis. *Megasphaera*, which was enriched in LTS PDAC patients, could produce SCFAs, such as butyrate, valerate, and isovalerate, and induce activation of Tc cells and tumor necrosis factor (TNF)‐α production by TAMs [69]. Butyrate‐producing bacteria, like *Finegoldia* and *Nocardioides*, which were also enriched in patients with LTS, could enhance intracellular lipid accumulation by increasing fatty acid transport and inhibiting lipolysis, and induce oxidative stress via SOD2 downregulation, thus triggering greater lipid peroxidation and ferroptosis in PDAC [70].

A recent single‐cell analysis of human PDAC showed that the upregulated immune signaling pathways in bacteria‐associated cells are related to the complement cascade and PD‐1 signaling, consistent with the results of preclinical studies [61]. Upregulated pathways are also enriched in cell motility, for example, MET–PTK2 signaling, related to cancer invasion and aggressiveness, as well as ECM interactions, involving impaired tissue integrity [61]. Moreover, this study revealed that some bacteria colocalized with tumor cells; specifically, *H. pylori*, *Francisella* spp., and *F. nucleatum* are respectively associated with increased expression of the oncogene CRABP2, the stem cell marker OLFM4, and REG3A, which induces acinar‐to‐ductal metaplasia, suggesting a direct procarcinogenic effect on tumor cells [61]. The aforementioned SRT study revealing the bacteria and CD8^+^ T cells coupling also showed that, despite enhanced cytolytic activity, humoral immunity, complement cascade, and inflammatory response compared with cold nests, the hot nests also displayed increased infiltration of MDSCs and Tregs and increased expression of PD‐L1 and T cell exhaustion markers [62]. The bacteria enriched in hot nests may help sustain immunological reactivity in the TME by maintaining the M2‐like phenotype of TAMs with elevated levels of major histocompatibility complex II molecules and CD206, as well as increased B cell infiltration [62]. In contrast, tumor cells in cold nests are more proliferative, with the regulation of genes enriched in hypoxia, glycolysis, cell cycle regulation, MYC targets, DNA repair, and epithelial‐to‐mesenchymal transition (EMT) [62]. The heterogeneous distribution and effects of intratumoral bacteria in PDAC add complexity to the application of microbial therapy.

### 4.5 Metastatic Cancer

As metastases account for a high proportion of adverse events and death, it is critical to define differences in taxonomic composition between metastases and primary tumors; the interactions among microbes, tumor cells, and the immune TME within metastases; and the microbial impact on treatment response. A pan‐cancer analysis of microbiota in metastatic tumor samples, obtained mainly from the liver, lymph node, and lung, showed that the taxonomic composition was impacted more by the anatomical location of metastases rather than by the type of primary cancer, which is consistent with prior in vivo studies [8, 176]. Metastases with high microbial diversity correlated positively with the activation of transforming growth factor β, EGFR, and TNF‐α signaling, pathways related to ECM remodeling, CAF infiltration, and the enrichment of antimicrobial peptides and various innate immune cells [176]. ECM remodeling driven by microbe‐induced increases in various MMPs and actin cytoskeleton‐remodeling factors has been investigated in a mouse model of breast cancer, and certain taxa have been identified as key players, including *Staphylococcus*, *Lactobacillus*, and *F. nucleatum* [8, 54].

Lung metastasis is a common occurrence in various types of tumors due to the extensive blood supply and immunologically distinctive environment. This makes the lung an important metastatic niche in which microbiota dysbiosis may influence metastatic progression through immune modulation. Le Noci et al. found that airway‐delivered aerosolized antibiotics or probiotics, such as *L. rhamnosus*, markedly reversed the microbiota‐induced permissive microenvironment for melanoma lung metastasis by reducing immune tolerance, characterized by decreased IL‐10‐producing Tregs and M2‐like macrophages, as well as increased maturation of APCs and activation of T cells and NK cells in lung tissue [45]. However, the effects of microbiota modulation in the metastatic lung appear to be highly context dependent. Cheng et al. reported the opposite finding: microbiota ablation impaired the γδ T17‐cell‐ and IL‐17A‐mediated immune surveillance and antitumor response in the lung, thus promoting lung metastasis [177]. The frequency and maturation of γδ T17 cells in lung tissue significantly decreased owing to a reduction in IL‐6 and IL‐23 expression, whereas IFN‐γ producing γδ T1 cells were not affected [177]. Interestingly, a possible mechanistic explanation for this complexity was provided by Yao et al., who reported that, in a breast cancer lung metastasis model, intracellular bacteria within circulating tumor cells were associated with a more immunosuppressive phenotype of tumor‐associated neutrophils, characterized by an enhanced MDSC‐like suppressive program and increased PD‐L1 expression, potentially through the cyclic GMP‐AMP synthetase (cGAS)–STING–IL‐17B pathway triggered by cytosolic double‐stranded DNA accumulation. By contrast, extracellular bacteria selectively promoted the expansion of antigen‐presenting neutrophils. These observations suggest that intracellular and extracellular bacteria may differentially shape neutrophil states and antitumor immunity, and that microbial ablation targeting these distinct compartments could yield different therapeutic consequences [178].

Taken together, these studies indicate that intratumoral microbiota in metastases should be regarded as a dynamic component of the metastatic niche rather than a passive bystander. Unlike primary tumors, metastatic lesions may require site‐specific microbiota‐oriented strategies because microbial effects are shaped by the immune and physiological context of the host organ. This raises the possibility that metastatic‐site microbial features could provide distinct opportunities for biomarker development and localized therapeutic intervention, including selective microbial modulation, engineered probiotics, or phage‐based approaches. Meanwhile, these findings underscore the need for caution when using broad microbiota‐depleting strategies, such as antibiotics, in efforts to reshape antitumor immunity.

### 4.6 Common Mechanistic Themes

While the role of intratumoral microbes in cancers beyond the aforementioned types has yet to be fully studied, their potential involvement is being increasingly explored, albeit with varying degrees of methodological constraints (Table 2) [82, 83, 84, 85, 86, 87, 88, 89, 90, 91, 92, 93, 94, 95, 96, 97, 98, 99, 100]. Overall, intratumoral microbial communities seem to converge on a few shared functional themes, including immune modulation, metabolic reprogramming, and activation of oncogenic or stress–response signaling pathways, as shown in Figure 3, while the direction and magnitude of their effects remain highly context dependent.

Immune modulation is a dominant and recurring mechanism. Intratumoral microbes can shape both innate and adaptive immunity by engaging PRRs, altering antigen presentation, and reprogramming cytokine and chemokine networks. Bacteria can activate TLR4–NF‐κB signaling in immune or tumor cells, thereby inducing autocrine/paracrine cascades among the tumor, immune and stromal compartments that collectively drive immune suppression [149, 171, 172]. Intratumoral fungi can also be recognized by PRRs such as Dectin‐1 and other CLRs, thereby modulating myeloid‐cell or ILC function and contributing to an immunosuppressive TME [81, 127, 173]. Bacteria‐derived HLA peptides are reported to be present in both tumor cells and immune cells and exhibit strong immunogenicity in melanoma [24]. In addition, microbiota‐derived toxins and metabolites can drive either proinflammatory signaling cascades or immunosuppressive TME remodeling, further shaping antitumor immunity in a context‐dependent manner [69, 150, 151, 152].

A second shared mechanism involves metabolic reprogramming of the tumor ecosystem. Tumor‐resident microbes, like *F. nucleatum*, may directly modulate host cell metabolism, for example by promoting extracellular lactate accumulation and glycolytic rewiring through the upregulation of GLUT1. Microbe‐derived metabolites can also influence tumor and immune cell states, thereby coupling microbial activity to TME metabolism [179]. Notably, the effects of microbes and their metabolites on tumors can be dual, either promoting or inhibiting tumor progression, depending on cancer type, concentration, and the TME [69, 70, 77]. For example, butyrate, a microbe‐derived SCFA, is generally considered tumor‐suppressive: it triggers oxidative stress, apoptosis or ferroptosis in tumor cells, enhances CD8^+^ T‐cell immunity, restrains Treg expansion, and/or reshapes dendritic‐cell function; such effects have been reported in intratumoral microbiota‐linked settings including melanoma and PDAC [70, 180, 181]. However, emerging evidence indicates that butyrate may exert cancer‐promoting effects in a concentration‐ and context‐dependent manner. At relatively low intratumoral levels in lung cancers, butyrate can epigenetically activate oncogenic programs and promote immunosuppressive M2‐polarization of macrophages, thereby facilitating tumor progression and metastasis [77, 128].

Third, intratumoral microbes can activate oncogenic and stress–response signaling pathways and/or induce epigenetic modulation within cancer cells. Oncogenic viruses, such as HPV, HBV, and EBV, have been well established to trigger host genetic mutation or instability, providing canonical examples of microbe‐driven tumorigenesis. Beyond viruses, intratumoral microbial burden in tumor cells is positively associated with activation of oncogenic signaling pathways, including Wnt‐β‐catenin, NF‐κB, and STAT3 signaling pathways, which are involved in cell proliferation, EMT, genomic instability, and invasive behavior [51, 155, 156, 157]. However, mechanistic dissection at the level of individual microbial species remains limited and is largely confined to a small number of well‐studied taxa, such as *F. nucleatum* in CRC or Gammaproteobacteria in pancreatic cancer [160, 182]. Emerging evidence further suggests that intratumoral microbes can influence tumor progression through epigenetic mechanisms, including histone modification, chromatin accessibility, and noncoding RNA regulation, thereby reshaping transcriptional programs without directly altering the host genome [77, 128].

Finally, it is also important to recognize that intratumoral microbial effects are not uniformly tumor promoting. In selected contexts, certain microbes have been reported to exert antitumor activity by enhancing host antitumor immunity or improving therapeutic responsiveness. For example, *Bifidobacterium* spp. have been reported to enhance innate and adaptive antitumor immunity and improve the efficacy of ICIs in preclinical models, supporting their potential use as therapeutic adjuncts, either alone or in engineered forms [30].

Collectively, these studies indicate that intratumoral microbiota is not merely a passive bystander but an active regulator of tumor biology, with effects that vary across cancer types and metastatic niches. Table 3 provides a summary of these mechanistic findings, organized by microbial species. This growing body of evidence also raises the possibility that intratumoral microbiota may influence not only tumor progression itself, but also the efficacy of anticancer therapies.

**TABLE 3:** Species‐specific mechanisms of intratumoral microbes across cancer types.

Microbiota	Mechanism	Cancer type	References
*Fusobacterium nucleatum*	Microsatellite instability (MSI) and CpG island methylator phenotype (CIMP)	Colorectal cancer	[66]
	FadA adhesin binds E‐cadherin → activates β‐catenin/Wnt signaling; ↑inflammatory cytokines		[138]
	Activates TLR4/MyD88/NF‐κB signaling → ↑proliferation; ↓apoptosis		[155]
	ADP‐heptose‐like metabolite → activates ALPK1/TIFA/NF‐κB signaling → upregulates BIRC3 and TNFAIP3 → antiapoptosis		[158]
	Activates NF‐κB signaling → elevates chemokines (e.g., IL‐8, CXCL1) → proinflammatory TME		[160]
	Fap2 protein binds TIGIT receptor on NK/T cells → inhibits immune cell killing	Colorectal, gastric cancers	[159]
	Fap2‐dependent binding → GM‐CSF, IL‐8, CXCL1, MIP‐3α → ↑proliferation and migration	Pancreatic ductal adenocarcinoma	[175]
	Fap2‐dependent binding → suppresses T cell accumulation; ↑MMP‐9	Breast cancer	[54]
Enterotoxigenic *Bacteroides fragilis* (ETBF)	BFT binds E‐cadherin → activates β‐catenin/Wnt signaling; ↑c‐MYC → ↑proliferation	Colorectal cancer	[148]
	Activates TLR4/NFAT5 signaling → induces histone demethylase 2B (JMJD2B); demethylates H3K9me3 → upregulates NANOG and SOX2 expression → maintains stemness of cancer stem cells		[149]
	Induces E‐cadherin/β‐catenin and Notch signaling pathway → increased stemness and invasiveness → ↑proliferation and metastasis	Breast cancer	[75]
*Escherichia coli* (pks^+^)	Colibactin toxin causes DNA double‐strand breaks and chromosomal instability	Colorectal cancer	[183]
*Helicobacter pylori*	Oncoprotein CagA perturbs host pathways (STAT3, NF‐κB, Hippo, Wnt); induces ROS, DNA damage, chronic inflammation	Gastric adenocarcinoma	[184]
*Porphyromonas gingivalis*	Elevates neutrophil chemokines (e.g., CXCL1/2) and neutrophil elastase (NE) → proinflammatory TAN‐rich microenvironment	Pancreatic ductal adenocarcinoma	[185]
*Salmonella Typhimurium*	Induces KDM6B (H3K27 demethylase) activation → epigenetic reprogramming and chronic inflammation	Gallbladder carcinoma	[186]
Clostridiales	Metabolite TMAO induces Caspase 3 and GSDME‐mediated pyroptosis → initiates endoplasmic reticulumstress → enhances the antitumor immune response and the efficacy of anti‐PD‐1 immunotherapy	Breast cancer	[25]
*Candida albicans*	Binds Dectin‐3 → reprograms macrophage metabolism toward glycolysis → IL‐17, IL‐22 secretion	Colorectal cancer	[164]
	Hyphae facilitate the penetration of *F. nucleatum* through a Flo9–RadD interaction		[165]
*Malassezia globosa/alternata*	Activates lectin (MBL) complement cascade → C3 → C3a binds C3aR on tumor cells (↑proliferation); also via Dectin‐1/CARD9 → IL‐33 → Th2/ILC2 recruitment	Pancreatic ductal adenocarcinoma	[22]
*Aspergillus sydowii*	β‐glucan induces Dectin‐1/CARD9 pathway → recruits MDSCs → IL‐1β secretion	Lung adenocarcinoma	[81]

*Abbreviations*: CagA, cytotoxin‐associated gene A; H3K27, histone H3 lysine 27; KDM6B, lysine demethylase 6B; MIP‐3α, macrophage inflammatory protein‐3α; MMP‐9, matrix metalloproteinase‐9; NE, neutrophil elastase; ROS, reactive oxygen species; TAN, tumor‐associated neutrophil; TIGIT, T‐cell immunoreceptor with Ig and ITIM domains.

## 5 Effects of Intratumoral Microbiota on Antitumor Treatment Efficacy

Given the increasingly recognized roles of intratumoral microbiota in cancer biology, their relevance may extend beyond tumor initiation and progression to the modulation of antitumor treatment efficacy. Accumulating evidence from both mechanistic and clinical studies suggests that intratumoral microbial features may influence treatment response, recurrence, metastasis, and patient survival [89]. In some clinical settings, intratumoral microbial signatures have also shown potential as predictive biomarkers of metastatic propensity and therapeutic outcomes. For example, in metastatic melanoma, a machine learning‐based study suggested that baseline intratumoral microbial features, particularly when integrated with host gene‐expression profiles, may improve the prediction of response to ICIs [187]. These findings support the emerging view that intratumoral microbiota may not only shape therapeutic sensitivity and resistance, but may also provide opportunities for biomarker development and microbiota‐targeted intervention. In this section, we summarize how intratumoral microbiota affects the efficacy of major anticancer therapies and discuss its potential translational implications.

### 5.1 Radiotherapy and Chemotherapy

Resistance to radiotherapy and chemotherapy is a major challenge in the treatment of various types of cancer, and several recent studies have suggested that microbe‐mediated resistance to both treatment modalities reduces antitumor efficacy.

PDAC ranks among the most lethal cancers, with widespread resistance to chemotherapy due to extensive desmoplastic stromal remodeling and poor vascularization [188, 189]. Gemcitabine (2′,2′‐difluorodeoxycytidine; GEM), used alone or in combination with nab‐paclitaxel, has emerged as a first‐line treatment for PDAC [188, 190]. Gammaproteobacteria are the most enriched class in human pancreatic tumors, and many species in the Enterobacteriaceae family can express the long form of the enzyme cytidine deaminase (CDD_L_), which is able to metabolize GEM into its inactive form (2′,2′‐difluorodeoxyuridine) and prevent GEM from inhibiting DNA synthesis in tumor cells [73, 191]. Ablation of either bacteria or fungi has been shown to augment the effect of GEM‐based chemotherapy in orthotopic PDAC models [22, 191]. Resistance to GEM mediated by the Enterobacteriaceae family has also been confirmed in CRC both in vitro and in vivo [73]. In addition, *F. nucleatum* can confer resistance to oxaliplatin in CRC by activating autophagy through innate immune TLR4/MyD88 signaling and thereby inhibiting apoptosis and was found at higher levels in tumor tissue of CRC patients with recurrence after chemotherapy [157]. *L. iners*, a common cervicovaginal commensal species, has been shown to confer resistance to chemoradiotherapy (CRT) in human cervical cancer via its obligate metabolite L‐lactate [80]. Intratumoral *L. iners* can increase the utilization and production of L‐lactate in response to oxidative and other metabolic stresses from CRT, rendering L‐lactate more available than glucose for the TCA cycle and contributing to glycolysis, galactose, and glutamate metabolism [80]. This L‐lactate‐mediated metabolic coupling between *L. iners*, tumor cells, and nontumor cells within the TME promotes energy production and DNA synthesis and enhances the Warburg effect, favoring rapid tumor cell proliferation [80]. In addition, *L. iners* strains derived from cervical cancer tissue can trigger G2/M checkpoint arrest via the Cdk1/Cyclin B complex, thereby enhancing tumor cell survival following radiation in vitro, which may be attributed to the extra *lacG* gene acquired during carcinogenesis [80].

Overall, targeting the intratumoral microbiome may provide a novel adjuvant strategy to reduce the prevalence of drug or radiation resistance. However, long‐term antibiotic treatment may induce antibiotic‐resistant microbes and suppress the microbiota at other sites, particularly in the gut, suggesting the need to explore appropriate routes of antibiotic administration or targeted drugs against effector molecules [171, 192, 193]. Interestingly, the structures or components of certain harmful intratumoral microbes can be used as vehicles to facilitate the accurate release of drugs in tumors. For instance, due to Fap2‐mediated specific binding to Gal–GalNAc overexpressed on breast tumors, the membrane of *F. nucleatum* was fused with liposomes to construct a superior tumor‐targeted vehicle. This vehicle was then loaded with colistin and doxycycline to eradicate the intratumoral microbiota, thereby improving chemotherapy efficacy and inhibiting lung metastasis in vivo [194]. Wu et al. fabricated a GalNAc‐derived nanoplatform that inhibits the adhesion and subsequent interaction of *F. nucleatum* with CRC cells, thus overcoming *F. nucleatum*‐mediated resistance to chemotherapy [195]. Moreover, photodynamic therapy shows promise as a spatiotemporally specific method for intratumoral microbiota eradication while minimizing the influence on the gut microbiota, for example, Liu et al. showed that an antimicrobial peptide conjugated with a photosensitizer could target the intratumoral microbiota and kill these bacteria through ROS‐induced membrane depolarization after irradiation in the tumor area, thereby overcoming the GEM resistance in PDAC [196].

### 5.2 Immunotherapy

The role of the microbiota in modulating the immune TME provides a rationale for combining microbiota‐based strategies with antitumor immunotherapy, particularly ICI treatment, across cancer types. Gut microbiota signatures have been extensively investigated in preclinical and clinical studies for their effects on the ICI response; for example, some commensal probiotics, such as *Bifidobacterium* species, *L. johnsonii*, and *Akkermansia muciniphila*, can boost ICI efficacy by enhancing innate and adaptive immune responses in patients with melanoma [197, 198, 199, 200, 201].

Recently, research on the effects of intratumoral microbiota on ICI treatment has expanded. *Bifidobacterium* colonizing CRC tumors can induce Type I IFN signaling via the STING signaling pathway and promote cross‐priming of DCs within the TME, which induces a rapid innate immune response and links innate and adaptive immunity, thereby facilitating anti‐CD47 treatment [202]. In addition, the intratumoral genus *Escherichia* was found to be associated with longer OS in advanced NSCLC patients receiving anti‐PD‐1/L1 treatment, probably due to an antitumor immune‐activated TME, characterized by upregulated expression of GZMB, CCL20, and CXCL13 in *Escherichia*‐positive tumor tissue [78]. Another study reported a higher proportion of *Sphingomonas* and *Pseudomonas* genera in NSCLC patients responding to combined chemotherapy and ICI treatment compared with nonresponders [79]. In contrast, a high abundance of *F. nucleatum* in metastases was associated with a poor response to ICI monotherapy [176]. Increasing the release of pathogen‐associated molecular patterns (PAMPs) by lysing intratumoral bacteria may be a novel and promising way to augment immunotherapy, as the significance of pattern recognition of intratumoral microbes has been widely demonstrated across cancer types. Liu et al. constructed an *F. nucleatum*‐derived outer membrane vesicle‐coated metronidazole‐loaded nanoplatform to target the tumor site, kill both the tumor cells and *F. nucleatum*, and increase the release of damage‐associated molecular patterns and PAMPs, contributing to DC maturation and thereby promoting the antitumor adaptive immune response [203]. Such a strategy facilitates the maintenance and reversal of the immunogenicity induced by intratumoral microbiota to maximize immunotherapy efficacy, particularly important for “cold” tumors.

Bender et al. showed that *L. reuteri* can translocate from the gut microbiota and colonize melanoma and metabolize dietary tryptophan into indole‐3‐aldehyde, which activates the aryl hydrocarbon receptor and promotes the proliferation and activation of IFN‐γ^+^CD8^+^ T (Tc1) cells via the induction of CREB‐mediated transcription, thus enhancing anti‐PD‐L1 efficacy [46]. *Megasphaera*, specifically enriched in the tumor tissues of PDAC patients with LTS, may facilitate anti‐PD‐1 treatment via its metabolites SCFAs in vivo, which can enhance the Tc cell function and the production of proinflammatory cytokines [69]. TMAO produced by Clostridiales could activate endoplasmic reticulum stress‐induced pyroptosis of tumor cells in TNBC, thus enhancing antitumor immune response and anti‐PD‐1 efficacy [25].

Immunotherapy could in turn reshape the microbial communities inside tumors. Battaglia et al. observed decreased microbial abundance and significant alterations in the taxonomic composition of metastases following immunotherapy, particularly in patients who responded favorably [176]. These changes were not observed after chemotherapy (except for antibiotic‐like agents like doxorubicin/epirubicin and bleomycin) or targeted therapy [176]. Several bacterial taxa, such as Bacteroidetes, *Actinomyces*, *Bifidobacterium*, and *Prevotella*, which were typically eliminated after ICI treatment, were found to have peptides previously identified as being presented on tumor cells via HLA [24]. Since the microbe‐derived peptides serving as neoantigens may play a critical role in maintaining antitumor adaptive immune response, gradual depletion of these microbes due to ICI treatment may result in reduced reactivity [167].

### 5.3 Engineered Bacteria

Due to the abundant intratumoral microbiota and tumor‐specific microbial signatures, reprogramming tumor‐targeting microbes to enhance antitumor immunity and/or kill tumor cells may be a promising therapeutic strategy to help eradicate tumor cells without damaging normal tissues. Certain “cold tumors,” such as malignant melanoma and TNBC, which exhibit low levels of tumor‐associated antigens and immune cell infiltration, are poorly responsive to immunotherapy, underscoring the importance of bacteria‐mediated cancer therapy [139, 204]. Prevalent regions of necrosis and hypoxia within solid tumors contribute to the colonization and enrichment of some obligate or facultative anaerobic bacteria, such as the genera Salmonella, Escherichia, and Listeria, which accumulate in tumor tissues at concentrations more than 1000‐fold higher than those in healthy tissues [205]. Several bacterial strains with attenuated virulence, increased tumor targeting and/or effector systems have been constructed for enhanced therapeutic effects and evaluated in vitro and in vivo (Table 4) [206, 207, 208, 209].

**TABLE 4:** Preclinical and clinical microbiota‐targeted therapeutic strategies in cancer.

Intervention category	Strategy	Cancer type	Phase of research	Status	Proposed mechanism	References
Microbial depletion/antibiotics	Metronidazole + chlorhexidine (CHX)	Oral squamous cell carcinoma	Phase II clinical trial	Recruiting	Preoperative antibiotic modulation to reduce intratumoral bacterial burden and assess associated changes in the immune microenvironment (e.g., T cells, neutrophils/polymorphonuclear myeloid‐derived suppressor cells [PMN‐MDSCs], cytokines)	NCT06627270
	Metronidazole	Locally advanced rectal adenocarcinoma		Recruiting	Metronidazole course + standard neoadjuvant therapy; exploratory analysis of immune and microbial infiltrates in pre‐ and posttreatment tumor tissues	NCT06569368
	Metronidazole	Colorectal cancer	Phase II/proof‐of‐concept clinical trial	Recruiting	Preoperative depletion of *Fusobacterium nucleatum* to reduce tumor colonization and evaluate effects on the TME	NCT05748145
Engineered bacteria	Attenuated *S. typhimurium* strain VNP20009	Lung cancer	Preclinical study	−	A combined therapy of antiangiogenic peptide HM‐3 and Sox2 ShRNA delivered by VNP20009	[210]
		Hepatocellular carcinoma and breast cancer			VNP20009 conjugated with aptamer	[211]
		Melanoma			Macrophage‐mediated delivery of VNP20009; expression of anti‐PD‐1 antibodies	[212]
		Metastatic melanoma and renal cell carcinoma	Phase I clinical trials	Completed (25 patients)	Intravenous (IV) infusion 10^6^ to 10^9^ CFU/m^2^ of VNP20009	[213]
		Advanced or metastatic solid tumors		Completed	Intratumoral (IT) injection escalating doses until the maximum tolerated dose (MTD) or the optimal biologic dose	NCT00004216
	Lipid A‐negative VNP20009	Colon cancer	Preclinical study	−	Synthesize the cytokine IL‐18	[214]
		Colon and lung cancer			Synthesize the cytokine LIGHT	[215]
	Attenuated *S. typhimurium* strain ΔppGpp	Colon cancer	Preclinical study	−	Carry cytolysin A to kill cancer cells	[216]
		Colon cancer			Carry cytolysin A to kill cancer cells in combination with radiotherapy	[209]
		Colon cancer and melanoma			Secrete heterologous *Vibrio vulnificus* flagellin B	[217]
		Breast cancer and melanoma			Carry the RGD peptide on the OmpA of ΔppGpp	[218]
	Attenuated *S. typhimurium* strain SL3261	B‐cell lymphoma	Preclinical study	−	Carry cGAS to stimulate STING signaling pathway	[219]
	Attenuated *S. typhimurium* strain BRD509	Melanoma	Preclinical study	−	Express IFN‐γ and trigger tumor cell killing	[220]
		Colon cancer and melanoma			Carry short hairpin (sh)‐INHA	[206]
	Attenuated *S. typhimurium* strain SL7207	Ovarian cancer	Preclinical study	−	Carry multidrug‐resistance gene (MDR1) small interfering (si) RNA	[207]
	Attenuated *S. typhimurium* strain	Unresectable hepatic metastases from nonhematologic primary solid tumors	Phase I clinical trials	Completed (22 patients)	Express IL‐2; oral administration, dose escalation to MTD	NCT01099631
	*E. coli* Nissle 1917 (EcN)	Colon cancer	Preclinical study	−	Convert tumor metabolic waste ammonia into L‐arginine, thus enhancing antitumor immune response	[221]
		Colorectal cancer			Facilitate the conversion of dietary glucosinolate to antitumor sulforaphane by secreting the enzyme myrosinase	[222]
		Liver cancer			Produce p53 and Tum‐5 within tumors	[223]
		B‐cell lymphoma and colorectal cancer			Stabilized intratumoral release of nanobodies targeting PD‐1 and CTLA‐4 using SLIC	[224]
		B‐cell lymphoma			Stabilized intratumoral release of nanobodies targeting CD47 using SLIC	[225]
		Solid tumors			Delivery of CAR targets to activate CAR‐T cells in situ using SLIC	[226]
		B‐cell lymphoma			Express anti‐CTLA‐4 or anti‐PD‐L1 nanobodies in response to focused ultrasound	[227]
		Breast cancer			Produce TNF‐α in response to thermal stimulation	[228]
		Solid tumors			Produce melanin and anti‐PD‐1 in response to laser irradiation	[229]
		Distant tumors			Enhance production of capsular polysaccharides for systemic delivery	[230]
		Metastatic solid tumors and lymphoma	Phase I clinical trials	Completed (32 patients)	A fumarate‐and‐nitrate reductase promoter (PfnrS)‐dacA circuit triggers STING signaling pathway in response to hypoxia; IT injection, dose escalation to MTD, alone and in combination with atezolizumab	[231]; NCT04167137
	Attenuated *E. coli* ClearColi BL21	Colon cancer	Preclinical study	−	Express anti‐CD47 nanobodies in response to magnetic signals	[232]
	*Bifidobacterium longum strain* NCC2705	Colon cancer	Preclinical study	−	Produce Tumstatin to inhibit tumor growth and angiogenesis	[208]
	Recombinant *Bifidobacterium longum* APS001F	Advanced and/or metastatic solid tumors	Phase I/II clinical trials	Completed (75 patients)	Produce cytosine deaminase; APS001F infusion in combination with flucytosine (5‐FC)	NCT01562626
	*Clostridium (C.) Novyi‐NT Spores*	Solid tumor malignancies	Phase I clinical trials	Completed (5 patients)	IV infusion, dose escalation, from 10^5^ to 10^7^ spores/kg	NCT01118819
		Treatment‐refractory solid tumor malignancies		Completed (2 patients)	IV infusion, dose escalation	NCT00358397
		Treatment‐refractory solid tumors		Completed (24 patients)	IT injection, dose escalation, from 10^4^ to 10^6^ spores/kg	NCT01924689
		Advanced solid tumor malignancies		Active, not recruiting	IT injection with dose escalation, in combination with pembrolizumab	NCT03435952
	Attenuated *Listeria monocytogenes* ADXS‐503	Metastatic NSCLC	Phase 1/2 clinical trials	Completed (24 patients)	1 × 10^8^ or 5 × 10^8^ CFU of ADXS‐503, alone and in combination with pembrolizumab	NCT03847519
	A genetically modified strain of *Yersinia Enterocolitica*, T3P‐Y058‐739	Advanced and unresectable solid tumour		Recruiting	IV infusion or IT injection, alone and in combination with pembrolizumab	NCT05120596
Probiotics	*Bifidobacterium infantis*‐based hypoxia‐targeted DOX nanoparticle delivery system	Breast cancer	Preclinical study	−	Deliver chemotherapeutic nanoparticles to hypoxic tumors, increasing intratumoral drug retention and antitumor efficacy	[233]
	*Bifidobacterium infantis* pretreatment combined with specific antibody	Lung cancer		−	Reduce tumor hypoxia and enhance radiosensitivity in combination with radiotherapy	[234]
	Gallium‐polyphenol‐functionalized *Lactobacillus rhamnosus* GG (LGG@Ga‐poly)	Pancreatic cancer		−	Modulate intratumoral microbiota, reduce intratumoral Proteobacteria/LPS, and enhance antitumor immunity	[235]

*Abbreviations*: 5‐FC, 5‐fluorocytosine; CFU, colony‐forming units; CHX, chlorhexidine; DOX, doxorubicin; INHA, inhibin subunit alpha; LGG, Lactobacillus rhamnosus GG; MDR1, multidrug resistance protein 1; MTD, maximum tolerated dose; PMN‐MDSCs, polymorphonuclear myeloid‐derived suppressor cells.

*Salmonella* (*S*.) *typhimurium* has been shown to activate immunity in the TME via its components LPS and flagellin, characterized by increased infiltration and activation of immune cells and release of TNF‐α, IL‐1β, and IFN‐γ. Moreover, intracellular *S. typhimurium* can kill tumor cells by inducing apoptosis and autophagy [210, 236]. The safety of using the attenuated *S. typhimurium* strain VNP20009, with *purI* and *msbB* deletions, has been confirmed in Phase I clinical trials for the treatment of metastatic melanoma and metastatic renal cell carcinoma, whereas the tumor colonization and antitumor efficacy are insufficient, probably due to the limited tumor targeting ability and dose‐dependent side effects [213]. Geng et al. found that aptamer‐conjugated VNP20009 can better colonize tumor tissue and stimulate a local immune response, exerting superior antitumor efficacy compared with VNP20009 alone [211]. Wu et al. recently designed a tumor‐targeting, macrophage‐mediated delivery system for VNP20009 engineered to express anti‐PD‐1 antibodies, which achieved strong antitumor efficacy and reduced toxicity in a melanoma mouse model [212]. A further attenuated ΔppGpp *S. typhimurium* strain induces increased infiltration of immune cells and shows a higher complete tumor eradication rate and longer survival in a mouse model of CRC compared with VNP20009 [237]. Engineering of ΔppGpp *S. typhimurium* with the arginine–glycine–aspartate (RGD) peptide on the outer membrane protein A (OmpA) promotes tumor targeting via the overexpressed αvβ3 integrin on tumor cells and new blood vessels and enhances antitumor effects [218, 238, 239]. Different engineered effector systems of *S. typhimurium* expressing cytolysin A, cytokines (e.g., IL‐2, IL‐18, IFN‐β, IFN‐γ, and human cytokine LIGHT), cGAS, β‐galactosidase, and *Vibrio vulnificus* flagellin B have demonstrated antitumor activity by remodeling the immune TME and/or directly killing tumor cells, without causing significant toxicity in mouse models [204, 214, 215, 216, 217, 219, 220, 240].

*E. coli* is also a well‐characterized species for cancer therapy [222, 223, 230, 241]. *E. coli* Nissle 1917 (EcN) and Symbioflor‐2 strains display superior tumor targeting and tumor specificity, but not equivalent antitumor potency [242]. Various genetic circuits, including effector elements and promoters or repressors, are introduced into EcN to enable it to sense and respond to internal and/or external triggering signals [243]. SYNB1891 is designed by incorporating the dacA gene from *Listeria monocytogenes* into the EcN genome and constructing a fumarate‐and‐nitrate reductase promoter (PfnrS)‐*dacA* circuit [231]. This circuit can be induced by the hypoxic TME to produce high levels of cyclic di‐AMP, which drives APC activation, Type I IFN production, and neoantigen cross‐presentation to Tc cells via STING pathway activation, thereby bridging innate and tumor‐specific adaptive immunity [231]. Compared with small‐molecule STING agonists, SYNB1891 activates the STING pathway in a phagocytosis‐dependent manner, preventing its “off‐target” activation in T cells, which has previously been found to be associated with the inhibition of immunological memory formation [231, 244]. The therapeutic efficacy of SYNB1891 alone or in combination with atezolizumab is being evaluated in an ongoing Phase I trial in patients with advanced solid tumors and lymphoma [231]. Engineered bacteria equipped with a synchronized lysis circuit (SLIC) represent a well‐researched strategy for achieving intratumoral quorum‐sensing‐driven bacterial lysis, enabling the release of therapeutic payload in a coordinated manner [243]. Using EcN containing a SLIC for the delivery of ICIs, such as anti‐PD‐L1, anti‐CTLA‐4, and anti‐CD47 nanobodies, has been shown to further enhance tumor regression, prevent metastasis and prolong survival compared with a SLIC‐negative strain expressing the corresponding ICIs or with intravenous injection in mouse models of multiple solid tumors [224, 225]. SLIC‐integrated EcN can also be used for the continuous conversion of the tumor metabolic waste product ammonia into L‐arginine within tumors, thereby increasing T cell infiltration and synergizing with anti‐PD‐L1 therapy [221]. Moreover, EcN has been engineered with a SLIC to deliver chimeric antigen receptor (CAR) targets to immune‐privileged tumor cores, providing antigen‐independent costimulatory signals for the activation of CAR‐T cells and CAR‐mediated lysis in situ, and can be safely administered for the treatment of multiple solid tumors [226]. Several engineered EcN strains are designed to respond to external physical signals, endowing them with spatiotemporal controllability and sustained in situ activity once stimulated [227, 228, 229, 232]. For example, Abedi et al. reprogrammed EcN with a repressor TcI42‐based thermal transducer and effector elements expressing anti‐CTLA‐4 or anti‐PD‐L1 nanobodies, allowing engineered EcN to respond robustly to tumor heating caused by noninvasive focused ultrasound [227].

Conversely, therapies that target tumor neovascularization could expand the hypoxic and necrotic regions within the solid tumor, thus facilitating the colonization of anaerobic engineered bacteria and enhancing the synergy between microbial therapy and cytotoxic therapy [205].

### 5.4 Probiotics

Probiotics have been extensively studied to facilitate ICI treatment by influencing the gut microbiota and modulating immunity [245, 246]. Compared with engineered bacteria, probiotics may serve as safer carriers for tumor‐targeted drug delivery and may also modulate the existing intratumoral microbiome. Anaerobic *Bifidobacterium infantis* could selectively target the poorly vascularized hypoxic area of breast tumors and deliver chemotherapeutic adriamycin nanoparticles in situ, improving the efficacy of antitumor therapy and reducing off‐target side effects [233]. Moreover, *B. infantis* itself could exert antitumor effects, consistent with previous studies [233, 234]. Gallium‐polyphenol network‐modified *L. rhamnosus* could specifically target PDAC and eliminate the Proteobacteria phylum, *E. coli*, and procarcinogenic *F. nucleatum* by disrupting iron respiration, which prevents TLR activation and subsequent immune suppression and tolerance, thereby suppressing tumor growth [235]. The reduced expression of PD‐L1 enables this probiotic to facilitate immunotherapy [235]. *L. rhamnosus*, serving as a vector, overcame the limited penetration of free antimicrobial agents into the dense ECM within PDAC [235].

### 5.5 Clinical Translation and Safety Considerations

Preclinical and early clinical studies have explored several microbiota‐targeted strategies in cancer, including microbial depletion/antibiotics, engineered bacteria, and probiotics (Table 4). These approaches reflect the growing translational interest in intratumoral microbiota, yet the field remains in its infancy. Currently, few clinical trials are designed to directly target intratumoral microbes; most human evidence derives from observational analyses of tumor samples or indirect modulation strategies such as antibiotic use, dietary regulation, or fecal microbiota transplantation. Notably, although these approaches intersect with intratumoral microbiota research, they are not yet optimized for selective manipulation of intratumoral microbial communities. Likewise, engineered bacteria and probiotic‐based strategies are often used as delivery platforms or multifunctional therapeutic modalities, with effects involving immune modulation, tumor‐cell killing, and/or microenvironmental remodeling.

Several factors currently constrain direct clinical translation. First, the extremely low microbial biomass within tumor tissues poses technical challenges for robust detection and reproducible therapeutic targeting. Second, causal relationships between specific intratumoral microbes and tumor progression remain incompletely defined, complicating efforts to distinguish functional drivers from passive bystanders. Third, direct modulation raises substantial safety and ethical concerns, including off‐target effects, unintended immune activation, and uncertain long‐term oncological outcomes. Antibiotic‐based modulation, while capable of reducing procarcinogenic taxa, carries potential risks of systemic off‐target effects across distal organs and immune compartments, potentially disrupting protective microbiota communities and leading to uncertain long‐term outcomes. Similarly, the clinical translation of engineered bacterial platforms is tempered by legitimate safety concerns surrounding live biotherapeutic products, including the risk of unintended bacterial dissemination, potential immunotoxicity, and the inherent heterogeneity of human tumors that may lead to variable therapeutic responses. These considerations underscore the critical need for rigorously designed controlled trials that systematically evaluate not only efficacy but also short‐ and long‐term safety profiles, optimal dosing regimens, and patient selection criteria.

Given these limitations, integrating microbiota‐targeted therapies as response modifiers, rather than standalone interventions, represents a more clinically viable path forward. By reprogramming host–microbe interactions within the TME, these approaches may optimize the efficacy of radio‐, chemo‐, and immunotherapy, overcome treatment resistance, and mitigate therapy‐related toxicity. To realize this potential, future efforts should prioritize longitudinal intratumoral microbiome profiling throughout disease progression. Such dynamic monitoring could serve as a companion diagnostic for biomarker‐guided patient stratification, inform real‐time dose adjustment, and help minimize risks such as microbial translocation in immunocompromised patients.

## 6 Conclusions and Prospects

Intratumoral microbiota has emerged as a biologically and clinically relevant component of the tumor ecosystem. Across cancer types, intratumoral microbial communities show context‐dependent patterns that may support diagnostic and prognostic applications and may represent an updated “hallmark.” Mechanistic studies increasingly converge on several recurrent functional themes, including immune modulation, metabolic rewiring, genome instability, epigenetic modification, and activation of carcinogenic pathways, suggesting that intratumoral microbes can shape tumor behavior through localized host–microbe interactions within the TME. Importantly, these effects are more plausibly driven by community‐level perturbations and convergent host pathways than by any single “universal” intratumoral pathogen. Emerging therapeutic strategies use antibiotics, engineered attenuated bacteria, and probiotics to modulate the intratumoral microbiota. These strategies can be used as adjuvants to improve responses to chemotherapy, radiotherapy, and immunotherapy.

Despite the progress made so far, current research remains limited. First, taxonomic identification is subject to bias because of the low biomass of intratumoral microbiota and technological limitations, including the selection of hypervariable regions for amplicon sequencing and the methods chosen to exclude interference from host cell reads. Second, the molecular mechanisms of intratumoral microbiota require further investigation. However, the complexity of microbial composition, the TME, and the intricate dynamic crosstalk between microbes and various cells present challenges. It remains inconclusive whether intratumoral microbiota serves as a precursor to or a consequence of tumorigenesis, owing to limited causal evidence linking microbes to cell fate. Microbes may exert cascading effects on tumor cells and the TME through multiple mechanisms across different phases of tumor initiation and development; a “driver and passenger” model has been widely accepted. Some microbes and/or their metabolites may play bidirectional roles across different tumor types, adding to the complexity of related research. Meanwhile, distinguishing the effects of gut microbiota perturbation from those of intratumoral microbiota remains challenging in preclinical studies.

From a translational perspective, current interventions should be viewed primarily as adjunctive strategies. Antibiotic‐based modulation may reduce specific protumorigenic taxa, yet systemic exposure introduces off‐target effects across organs and immune compartments, requiring careful risk‐benefit assessment. Engineered bacteria and probiotic‐based platforms offer tumor tropism and programmable payload delivery, but clinical translation remains limited, in part due to safety concerns for live microbial products and the heterogeneity of human tumors.

Future progress will likely depend on integrating multiomics profiling with mechanistic validation and early‐phase clinical testing. Candidate opportunities include microbial or microbe‐derived biomarkers for stratification and monitoring, programmable bacterial vectors for tumor regions, and microbe‐associated antigens that may expand immunotherapy targets in poorly immunogenic tumors. Specifically, microbes along with their components and metabolites in the circulation and other body fluids could reflect the intratumoral microbiota composition and hold promise as potential noninvasive biomarkers. The genetic cargo capacity of bacteria allows the sustained delivery of therapeutic payloads, and the integration of various regulatory elements into their genomes can enable controlled release through either autonomous or external regulation, making engineered bacteria sophisticated vectors for increased temporal and spatial specificity. Engineered bacteria, typically well‐characterized anaerobic species, can target and colonize the hypoxic and necrotic regions of solid tumors, where systemically delivered drugs have limited access and low oxygen levels compromise the effectiveness of radiotherapy and hinder immune cell function during immunotherapy. The distinct regions targeted by microbial and cytotoxic therapies make engineered bacteria promising adjuvants for improved treatment outcomes. In addition, with respect to the intracellular microbes within tumors, several studies have uncovered microbe‐derived antigens present on the surface of tumor cells, which may serve as potential targets to enhance immunotherapy efficacy, particularly in cancers with low immunogenicity, such as TNBC and melanoma. Recently, specific innocuous structures or components of harmful intratumoral microbes, such as outer membranes, have been investigated as potential drug delivery systems, offering significant tumor‐targeting capabilities. With improved mechanistic resolution, validated signatures, and carefully designed clinical studies, intratumoral microbiota may ultimately become a practical lever for precision diagnostics and therapy optimization.

## Author Contributions

RJ, YZ, and YW supervised the organization and preparation of this review. MX prepared the original draft, including writing and visualization. JY and YC reviewed and edited the manuscript. SG contributed to conceptualization and review. LY contributed to language editing and proofreading of the manuscript. All authors read and approved the final manuscript.

## Funding

This work was supported by the National Natural Science Foundation of China (12405389, U23A20466), the National Ten Thousand Talent Programme (SQ2022RA2C000263), State Key Laboratory of Eye Health, the Innovative Research Team of High‐level Local Universities in Shanghai (SHSMU‐ZDCX20210900, SHSMU‐ZDCX20210902), Noncommunicable Chronic Diseases‐National Science and Technology Major Project (2023ZD0511000), Science and Technology Commission of Shanghai (24YF2723800), Shanghai Pujiang Program (24PJD052), Postdoctoral Science Foundation of China (2025M772377), and Sanming Project of Medicine in Shenzhen (No. SZSM202411008).

## Ethics Statement

The authors have nothing to report.

## Conflicts of Interest

The authors declare no conflicts of interest.

## Data Availability

The authors have nothing to report.
